# Dokdolipids A−C, Hydroxylated Rhamnolipids from the Marine-Derived Actinomycete *Actinoalloteichus hymeniacidonis*

**DOI:** 10.3390/md17040237

**Published:** 2019-04-20

**Authors:** Byeoung-Kyu Choi, Hwa-Sun Lee, Jong Soon Kang, Hee Jae Shin

**Affiliations:** 1 Department of Marine Biotechnology, University of Science and Technology, 217 Gajungro Yuseong-gu, Daejeon 34113, Korea; choibk4404@kiost.ac; 2 Marine Natural Products Chemistry Laboratory, Korea Institute of Ocean Science and Technology, 385 Haeyang-ro, Yeongdo-gu, Busan 49111, Korea; hwasunlee@kiost.ac; 3 Laboratory Animal Resource Center, Korea Research Institute of Bioscience and Biotechnology, 30 Yeongudanjiro, Cheongju 28116, Korea; kanjon@kribb.re.kr

**Keywords:** rhamnolipids, biosurfactants, *Actinoalloteichus hymeniacidonis*, rhamnose, cytotoxicity

## Abstract

Three new hydroxylated rhamnolipids, dokdolipids A−C (**1**−**3**) were obtained from the marine actinomycete *Actinoalloteichus hymeniacidonis*, which was isolated from a sediment sample collected off the coasts of Dokdo island, Republic of Korea. The structures of the isolated compounds were elucidated on the basis of 1D and 2D NMR and mass spectrometric data analyses. Their absolute configurations were assigned using the modified Mosher’s method and specific rotation values, as well as acid hydrolysis, chemical derivatizations and subsequent HPLC analysis to determine the configuration of the sugar moieties. All new compounds were evaluated for their cytotoxicity against six cancer cell lines, HCT-15, NUGC-3, NCI-H23, ACHN, PC-3 and MDA-MB-231. Compounds **1**−**3** displayed moderate cytotoxicity against all the cell lines tested with IC_50_ values ranging from 13.7−41.5 µM.

## 1. Introduction

Rhamnolipids belong to a class of biosurfactants composed of rhamnose linked to β-hydroxylated fatty acid chains [1]. Rhamnolipids are classified as mono-rhamnolipids, which contain a single rhamnose molecule and di-rhamnolipids, which contain two rhamnose sugar rings [2]. These biosurfactants are mainly produced by *Pseudomonas* species [3] such as *P. aeruginosa* [4], *P. chlororaphis*, *P. plantarii*, *P. putida*, and *P. fluorescens* [5]. Over the past three decades, rhamnolipids have been broadly investigated and extensively reviewed due to their biodegradability and reduced toxicity compared to synthetic surfactants as well as their various applications [6,7,8]. It has been widely recognized that rhamnolipids have surface active properties such as emulsification, dispersion, foaming, detergency, wetting and stabilization [9]. Moreover, various researchers have demonstrated that rhamnolipids display low toxicity, antimicrobial activities and the ability to suppress the growth of breast cancer cells [10,11]. These unique and diverse properties make them suitable to be used in a wide range of industrial demands such as the bioremediation of pollutants, cosmetics, food, pharmaceuticals and therapeutics [12].

**Figure 1:**
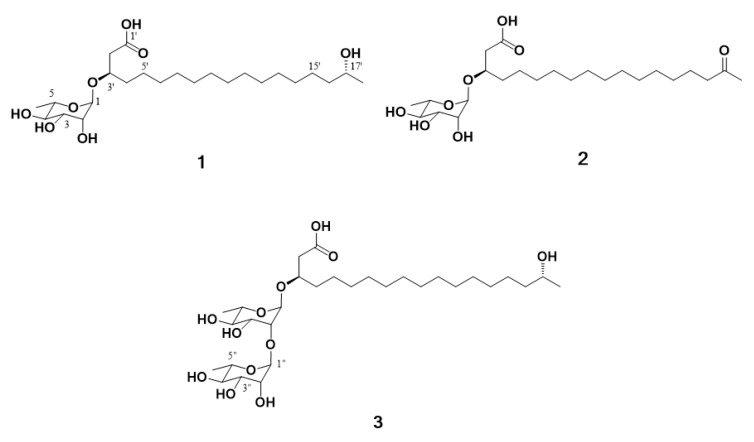
Structures of compounds **1**−**3** isolated from *Actinoalloteichus hymeniacidonis*.

The marine environment constitutes a significant reservoir of natural products which has offered the potential for new drug development over the last few decades [13]. Specifically, marine microorganisms are considered efficient producers of lead compounds with biomedical potential [14]. In addition, structurally diverse and impressive bioactive natural products have been identified from marine microbes [15]. In our continuous search for secondary metabolites from marine-derived bacteria, the *Actinoalloteichus hymeniacidonis* strain 179DD−027 was isolated from a deep-sea sediment sample collected off the coasts of Dokdo Island, East Sea, Republic of Korea. Dokdo Island is a large volcanic island with 89 small islets and rocks containing rich and well-preserved biodiversity [16]. Subsequent fermentation of the producing strain, solvent extraction and chemical investigation procedures led to the isolation of three new rhamnolipids, named dokdolipids A−C (**1**−**3**) (Figure 1). Dokdolipids represent the first rhamnolipids containing a hydroxyl (**1** and **3**) group and a ketone group (**2**) in the side chains. In this paper, we describe the isolation, structure elucidation and bioactivities of dokdolipids A−C (**1**−**3**).

## 2. Results and Discussion

**Figure 2:**
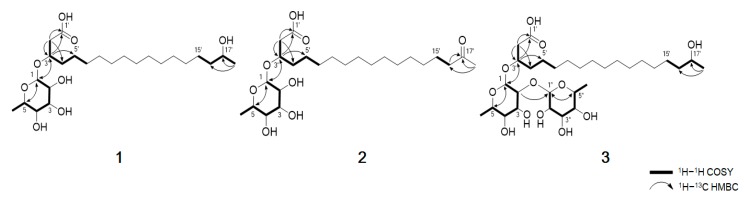
Key COSY and HMBC correlations of **1**−**3**.

Compound **1** was obtained as a dark brown oil and gave a [M + Na]^+^ ion peak at *m/z* 485.3094 (calcd 485.3090) in the HRESIMS, consistent with a molecular formula C_24_H_46_O_8_. The ^1^H NMR spectrum of **1** showed the signals of seven oxygenated methines (*δ*_H_ 4.80, 4.08, 3.75, 3.70, 3.65, 3.60 and 3.35), 14 methylene protons (*δ*_H_ 2.49, 1.55 and 12 overlapped protons at 1.29–1.45) and two methyls (*δ*_H_ 1.23 and 1.13). The ^1^H and ^13^C NMR data, in conjunction with HSQC of **1,** supported the presence of 24 carbons, which can be classified as one carbonyl (*δ*_C_ 173.9), seven sp^3^ methines (*δ*_C_ 98.9, 75.2, 72.5, 71.2, 70.9, 68.7 and 67.1), 14 sp^3^ methylene (*δ*_C_ 40.0–24.5) and two sp^3^ methyl (*δ*_C_ 22.0 and 16.2) carbon (Table 1). The planar structure of compound **1** was elucidated by analyzing the 2D NMR data, including the ^1^H–^1^H COSY and ^1^H–^13^C HMBC spectra (Figure 2). The COSY correlation from H_2_-2′ to highly overlapped proton signals, a terminal methyl group and methylene carbons at *δ*_C_ 24.5-29.4 suggested the presence of an aliphatic chain. The HMBC correlations from H_2_-2′ (*δ*_H_ 2.48, 2.53) to C-1′ (*δ*_C_ 173.9), C-3′ (*δ*_C_ 74.2) and C-4′ (*δ*_C_ 33.1) and from H-3′ (*δ*_H_ 4.08) to C-1′ (*δ*_C_ 173.9), C-2′ (*δ*_C_ 40.0) and C-5′ (*δ*_C_ 24.5) established the position of the carbonyl carbon C-1′ at *δ*_C_ 173.9 and the secondary alcohol H-3′ at *δ*_H_ 4.08. In addition, the chemical shift value of H-17′ (*δ*_H_ 3.70) and the HMBC correlation from the methyl doublet H_3_-18′ (*δ*_H_ 1.13, d, *J* = 6.2 Hz) to C-17′ (*δ*_C_ 67.1) and C-16′ (*δ*_C_ 38.8) indicated that a hydroxyl group was attached to C-17′. Detailed analysis of the 2D NMR spectra revealed the presence of a linear hydroxylated and saturated fatty acid as a 3, 17-dihydroxyoctadecanoic acid. Another spin system was identified from the H-1/H-2/H-3/H-4/H-5/H_3_-6 COSY correlations. A hexose moiety was confirmed by H-1/C-5 and H-5/C-1 HMBC correlations. The hexose ring was connected to C-3′ through an ether linkage which was confirmed by the H-3′/C-1 and H-1/C-3′ HMBC correlations. Thus, the planar structure of dokdolipid A (**1**) was elucidated as a new rhamnolipid.

**Figure 3:**
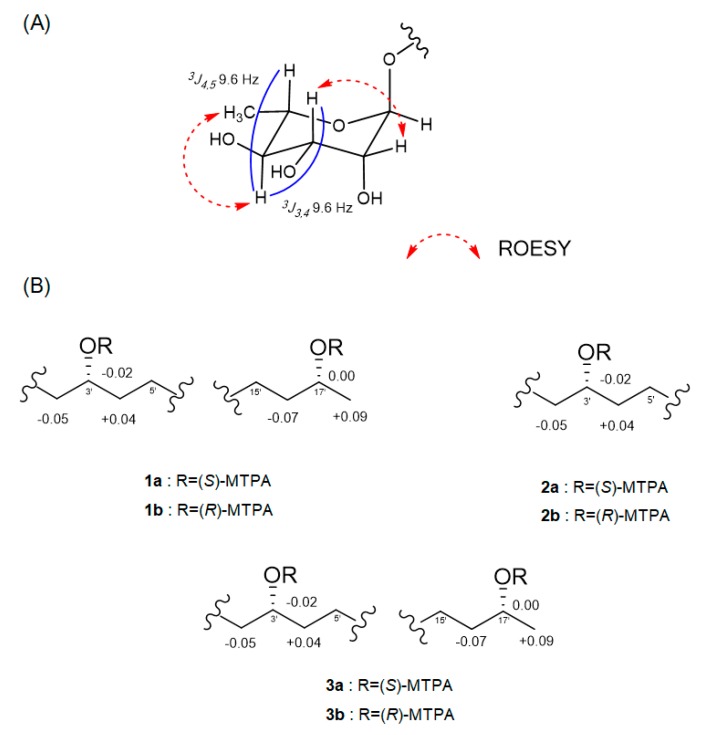
(**A**) ROESY correlations in the sugar moiety of **1.** (**B**) Δ*δ_S_*_−*R*_ values in ppm of the MTPA esters of the methylated aglycones of **1**–**3**.

The relative configuration of the sugar moiety was established by analyzing the vicinal coupling constant ^3^*J*_HH_ values and ROESY correlations, as seen in Figure 3A. The axial positions of H-3, H-4 and H-5 were assigned by the large coupling constants (^3^*J*_3,4_ = 9.6 Hz and ^3^*J*_4,5_ = 9.6 Hz). The broad singlet of the anomeric proton (H-1) and the ROESY correlations from H-2 to H-3 and from H-4 to H_3_-6 also suggested that the sugar moiety was rhamnose. To determine the absolute configuration, dokdolipid A (**1**) was subjected to a chemical degradation. The acid hydrolysis in MeOH of **1** afforded the methylated aglycone of **1** and rhamnopyranose. The absolute configuration of the methylated aglycone was confirmed using the modified Mosher’s method [17]. The observed chemical shift differences Δ*δ_S−R_* suggested the 3′*R*,17′*R* configurations in **1** (Figure 3B). In addition, the rhamnose obtained from hydrolysis and the authentic l- and d-rhamnose were separately derivatized with l-cysteine methyl ester hydrochloride and σ-Tolyl isothiocyanate to establish the absolute configuration of rhamnose [18]. On the basis of the HPLC analysis of the derivatives, the l-rhamnose moiety in **1** was confirmed by chemical derivatization and comparison with standards. Thus, the structure of **1** was identified to be (3′*R*,17′*R*)-3′-*O*-(α**-**l-rhamnopyranosyl)-17′- dihydroxyoctadecanoic acid and named as dokdolipid A (**1**).

Compound **2** was isolated as a dark brown oil, and its molecular formula was determined as C_24_H_44_O_8_ by the [M + Na]^+^ ion peak at *m/z* 483.2936 (calcd 483.2934) in the HRESIMS. The ^1^H and ^13^C NMR spectra of **2** were similar to those of **1**, suggesting that **2** shared the same carbon skeleton as **1**. The obvious differences were the disappearance of a doublet methyl and appearance of a singlet methyl. In addition, a ^13^C NMR signal of a carbonyl carbon at *δ*_C_ 210.8 was observed. The HMBC correlations from H_3_-18′ (*δ*_H_ 2.12) to C-16′ (*δ*_C_ 42.9) and C-17′ (*δ*_C_ 210.8) and from H_2_-16′ (*δ*_H_ 2.47) to C-17′ (*δ*_C_ 210.8) suggested that the oxygenated methine C-17′ in **1** was replaced by the carbonyl carbon in **2**. The absolute configuration of C-3′ in **2** was confirmed by the same method as that of **1,** as seen in Figure 3B. The results indicated the *R*-configuration of C-3′ in **2**. The comparison of the specific rotation values of **1** and **2** ([α]_D_^25^ −33.3 (c 0.3, MeOH) and [α]_D_^25^ −10.0 (c 0.3, MeOH), respectively) and identical chemical shifts also supported that **2** had the same absolute configuration as **1**. Thus, the structure of **2** was determined as a new derivative of **1** and named dokdolipid B (**2**).

Compound **3** was purified as a dark brown oil and gave a [M + Na]^+^ ion peak at *m/z* 631.3669 (calcd 631.3669) in the HRESIMS, consistent with a molecular formula C_30_H_56_O_12_. Comparison of the NMR spectroscopic data of **3** with **1** revealed that **3** has a very similar structure to that of **1**. However, the singlet at *δ*_H_ 1.23 corresponded to two methyl groups while the proton signal at *δ*_H_ 4.79 belonged to two anomeric protons. In addition, the presence of eight methine protons at *δ*_H_ 3.35–3.72 suggested that **3** possessed two hexose units. Further analysis of its 2D NMR data and coupling constants ^3^*J*_HH_ confirmed that the structure of **3** was analogous to that of **1** with two rhamnoses. The absolute configurations of C-3′ and C-17′ in **3** were also determined using the same method as **1** and the comparison of the specific rotation values of **1** and **3,** as well as similar chemical shifts, as seen in Figure 3B. By considering all the experimental data and the biosynthetic pathway of **1** and **3**, the absolute configuration of **3** was determined to be the same as **1**. Thus, the structure of **3** was elucidated and named dokdolipid C (**3**).

Compounds **1**–**3** were tested for their cytotoxicity against cancer cell lines including HCT-15, NUGC-3, NCI-H23, ACHN, PC-3 and MDA-MB-231 using sulforhodamine B (SRB) assay, with adriamycin as a positive control. As shown in Table 2, **1**−**3** showed moderate activity against these cells, with GI_50_ values ranging from 13.7 to 41.5 μM. Among the tested compounds, **2** displayed the strongest cytotoxicity in all the cell lines except for MDA-MB-231 (Breast cancer), whereas **3** showed better activity against the MDA-MB-231 cell line than other compounds.

## 3. Materials and Methods

### 3.1. General Experimental Procedures

The 1D (^1^H and ^13^C) and 2D (COSY, ROESY, HSQC, and HMBC) NMR spectra were acquired on a Bruker 600 MHz spectrometer. UV spectra were obtained on a Shimadzu UV-1650PC spectrophotometer. IR spectra were recorded on a JASCO FT/IR-4100 spectrophotometer. Optical rotations were measured on a Rudolph Research Analytical (Autopol III) polarimeter. HRESIMS spectra were recorded on a hybrid ion-trap time-of-flight mass spectrometer (Shimadzu LC/MS-IT-TOF). HPLC was performed on a PrimeLine Binary pump with RI-101 (Shodex). Analytical HPLC was conducted on an ODS column (YMC-Pack-ODS-A, 250 × 4.6 mm i.d, 5 µm).

### 3.2. Isolation and Cultivation of the Strain 179DD-027 (Actinoalloteichus hymeniacidonis)

The strain 179DD-027 was isolated from a sediment sample, collected off the coasts of Dokdo island, Republic of Korea. The strain was identified as *Actinoalloteichus hymeniacidonis* on the basis of the 16s rRNA gene sequence analysis (GenBank accession number MH681580). The strain 179DD-027 was grown on a Bennett’s (BN) agar plate for 7 days at 28 °C and then incubated in BN medium (composed of 10 g of glucose, 1 g of yeast extract, 2 g of tryptone, 1 g of beef extract, 5 g of glycerol and 32 g of NaCl in 1 L of H_2_O) in a 50 mL flask. After a four-day cultivation at 28 °C with shaking at 130 rpm, 10 mL of the seed culture in a 50 mL flask was used to inoculate 1 L of the culture medium in a 2 L flask for four days. For mass culture, 1 L of the culture in a 2 L flask was used to inoculate 40 L cultivation in BN medium in a 100 L fermenter. A total of 40 L of bacterial culture was incubated at 28 °C for 7 days.

### 3.3. Isolation of Compounds

The culture broth (40 L) was separated into cells and supernatant by centrifugation. The supernatant was extracted with EtOAc (40 L × 2) at room temperature and then concentrated under reduced pressure to yield the crude extract (3 g). The crude extract was fractionated by flash column chromatography on ODS using a stepwise elution (each fraction 300 mL × 3) with combinations of MeOH/H_2_O (1:4, 2:3, 3:2, 4:1 and 100% MeOH). The second fraction eluted with MeOH/H_2_O (4:1) was purified by an analytical, reversed-phase HPLC (YMC-Pack-ODS-A, 250 × 4.6 mm i.d, 5 *µ*m, flow rate 2.0 mL/min, RI detector) using isocratic elution with 40% MeCN in H_2_O to yield **1** (35.2 mg, *t*_R_ = 14 min), **2** (4.5 mg, *t*_R_ = 20 min), and **3** (5.8 mg, *t*_R_ = 9 min).

### 3.4. Spectral Data

Dokdolipid A(**1**): dark brown oil; [α]_D_^25^ −33.3 (c 0.3, MeOH); IR ν_max_ 3345, 2918, 2851, 1710, 1646, 1127, 1049 cm^−1^; UV(MeOH) λ_max_ (log ε) 318 (3.09), 218 (3.42) nm; HRESIMS *m/z* 485.3094 [M + Na]^+^ (calcd for 485.3090, C_24_H_46_O_8_Na); ^1^H NMR (CD_3_OD, 600 MHz) and ^13^C NMR (CD_3_OD, 125 MHz) see Table 1. 

Dokdolipid B (**2**): dark brown oil; [α]_D_^25^ −10.0 (c 0.3, MeOH); IR ν_max_ 3377, 2910, 2851, 1710, 1371, 1068, 1017 cm^−1^; UV(MeOH) λ_max_ (log ε) 406 (3.29), 312 (3.50), 238 (3.60) nm; HRESIMS *m/z* 483.2936 [M + Na]^+^ (calcd for 483.2934, C_24_H_44_O_8_Na); ^1^H NMR (CD_3_OD, 600 MHz) and ^13^C NMR (CD_3_OD, 125 MHz) see Table 1.

Dokdolipid C (**3**): dark brown oil; [α]_D_^25^ −40.0 (c 0.3, MeOH); IR ν_max_ 3693, 3328, 2971, 2858, 1632, 1349, 1058, 1010 cm^−1^; UV(MeOH) λ_max_ (log ε) 310 (3.15), 216 (3.51) nm; HRESIMS *m/z* 631.3669 [M + Na]^+^ (calcd for 631.3669, C_30_H_56_O_12_Na); ^1^H NMR (CD_3_OD, 600 MHz) and ^13^C NMR (CD_3_OD, 125 MHz) see Table 1.

### 3.5. Acid Hydrolysis and Determination of Absolute Configuration of Rhamnose

Each of the dokdolipids A−C (**1**−**3**) was dissolved in 3 N HCl (0.5 mL) in methanol and heated to 100 °C for 30 min. The solution was cooled and extracted with EtOAc twice. The EtOAc layer and the aqueous layer gave a methylated aglycone and a sugar residue after removal of the solvent respectively. The sugar residue was dissolved in pyridine (0.5 mL) containing l-cysteine methyl ester hydrochloride (0.5 mg) and heated to 60 °C for 1 h. σ-Tolylisothiocyanate (10 μL) was added to the mixture and heating was continued for an additional 1 h. The reaction mixture was directly analyzed using HPLC (10 to 100% MeCN gradient with 0.1% formic acid over 40 min). The sugar residue was detected at 17.9 min. The retention times of the authentic rhamnose samples were 15.5 (d-rhamnose) and 17.9 (l-rhamnose) min under the same HPLC conditions. Therefore, the absolute configuration of the rhamnose unit was established as l-configuration. All dokdolipids were also assigned using the chemical derivatization and HPLC analysis as described above. 

### 3.6. Preparation of MTPA and Esters of **1**−**3** using the Modified Mosher’s Method

(*R*)-MTPA-Cl (10 μL) or (*S*)-MTPA-Cl (10 μL) and anhydrous pyridine (200 μL) were added to a methylated aglycone (0.6 mg for each). The mixture was stirred overnight at room temperature. The reaction mixture was evaporated to dryness and extracted with EtOAc twice. The EtOAc extracts were purified using an analytical reversed-phase HPLC (YMC-Pack-ODS-A, 250 × 4.6 mm i.d, 5 µm, flow rate 2.0 mL/min, RI detector) using gradient elution from 70% to 100% MeOH in 40 min to yield **1a** (0.4 mg, *t*_R_ = 33 min) and **1b** (0.5 mg, *t*_R_ = 33 min). Using the same procedure, **2a** (0.3 mg, *t*_R_ = 27 min), **2b** (0.3 mg, *t*_R_ = 28 min), **3a** (0.3 mg, *t*_R_ = 32 min) and **3b** (0.4 mg, *t*_R_ = 33 min) were prepared from dokdolipids B and C (**2** and **3**, 1.0 mg for each), respectively.

Compound **1a**: ^1^H NMR (CD_3_OD, 600 MHz) *δ*_H_ 7.52−7.42 (10H, m, aromatic), 5.49 (1H, m, H-3′), 5.13 (1H, m, H-17′), 3.57 (3H, s, OMe), 3.55 (3H, s, OMe), 3.50 (3H, s, OMe), 2.64 (2H, dd, H-2′), 1.67 (2H, m, H-4′), 1.54 (2H, m, H-16′), 1.35−1.17 (22H, m), 1.32 (3H, d, H-18′); ESIMS *m/z* 785.2 [M + Na]^+^ (Supporting information).

Compound **1b**: ^1^H-NMR (CD_3_OD, 600 MHz) *δ*_H_ 7.52−7.42 (10H, m, aromatic), 5.46 (1H, m, H-3′), 5.13 (1H, m, H-17′), 3.66 (3H, s, OMe), 3.53 (3H, s, OMe), 3.52 (3H, s, OMe), 2.69 (2H, dd, H-2′), 1.63 (2H, m, H-4′), 1.61 (2H, m, H-16′), 1.35−1.17 (22H, m), 1.23 (3H, d, H-18′); ESIMS *m/z* 785.4 [M + Na]^+^ (Supporting information).

Compound **2a**: ^1^H NMR (CD_3_OD, 600 MHz) *δ*_H_ 7.52−7.42 (10H, m, aromatic), 5.50 (1H, m, H-3′), 3.57 (3H, s, OMe), 3.50 (3H, s, OMe), 2.64 (2H, dd, H-2′), 1.70 (2H, m, H-4′), 2.47 (2H, t, H-16′), 1.35−1.17 (22H, m), 2.12 (3H, s, H-18′); ESIMS *m/z* 567.4 [M + Na]^+^ (Supporting information).

Compound **2b**: ^1^H NMR (CD_3_OD, 600 MHz) *δ*_H_ 7.50−7.42 (10H, m, aromatic), 5.47 (1H, m, H-3′), 3.66 (3H, s, OMe), 3.53 (3H, s, OMe), 2.70 (2H, dd, H-2′), 1.62 (2H, m, H-4′), 2.47 (2H, t, H-16′), 1.35−1.17 (22H, m), 2.12 (3H, s, H-18′); ESIMS *m/z* 567.3 [M + Na]^+^ (Supporting information).

Compound **3a**: ^1^H NMR (CD_3_OD, 600 MHz) *δ*_H_ 7.52−7.42 (10H, m, aromatic), 5.49 (1H, m, H-3′), 5.13 (1H, m, H-17′), 3.57 (3H, s, OMe), 3.55 (3H, s, OMe), 3.50 (3H, s, OMe), 2.64 (2H, dd, H-2′), 1.70 (2H, m, H-4′), 1.55 (2H, m, H-16′), 1.35−1.17 (22H, m), 1.33 (3H, d, H-18′); ESIMS *m/z* 785.5 [M + Na]^+^ (Supporting information).

Compound **3b**: ^1^H NMR (CD_3_OD, 600 MHz) *δ*_H_ 7.52−7.42 (10H, m, aromatic), 5.46 (1H, m, H-3′), 5.13 (1H, m, H-17′), 3.66 (3H, s, OMe), 3.53 (3H, s, OMe), 3.52 (3H, s, OMe), 2.69 (2H, dd, H-2′), 1.66 (2H, m, H-4′), 1.60 (2H, m, H-16′), 1.35−1.17 (22H, m), 1.24 (3H, d, H-18′); ESIMS *m/z* 785.2 [M + Na]^+^ (Supporting information).

### 3.7. Cytotoxicity Test by SRB Assay

Human cancer cell lines HCT-15 (colon), NUGC-3 (stomach), NCI-H23 (lung), ACHN (renal), PC-3 (prostate) and MDA-MB-231 (breast), were purchased from the American Type Culture Collection (Manassas, VA). The cell lines were cultured RPMI 1640 supplemented with 10% fetal bovine serum (FBS). Cell cultures were maintained at 37 °C under a humidified atmosphere of 5% CO_2_. The growth inhibition assay against human cancer cell lines was carried out according to a sulforhodamine B (SRB) assay [19]. In brief, 8000 cells/well were seeded in a 96-well plate. Next day, the cells were treated with compounds **1**–**3** including vehicle control (0.1% DMSO) and positive control (adriamycin). After being incubated for 48 hours, cultures were fixed with 50% trichloroactetic acid (50 μg/mL) and stained with 0.4% sulforhodamine B in 1% acetic acid. Unbound dye was removed by washing with 1% acetic acid, and protein-bound dye was extracted with 10 mM Tris base (pH 10.5) for determination of optical density. The absorbance at 540 nm was determined using a VersaMax microplate reader (Molecular Devices, LLC, Sunnyvale, CA, USA). GI_50_ values were calculated using GraphPad Prism 4.0 software (GraphPad Software, Inc., San Diego, CA, USA).

## 4. Conclusions

In conclusion, the chemical analysis of the marine bacterium *Actinoalloteichus hymeniacidonis* 179DD-027 led to the isolation of three new hydroxylated rhamnolipids (**1**−**3**), and the identification of the sugar moiety as well as the determination of the absolute configuration of the stereogenic carbon in the carboxylic acid moiety. Dokdolipids A (**1**) and C (**3**) possess a hydroxyl group at C-17′ with one rhamnose and two rhamnoses respectively. Dokdolipid B (**2**) contains a ketone group at C-17′ with one rhamnose. To the best of our knowledge, this is the first report describing rhamnolipids with the hydroxyl and ketone groups at the fatty acid chain. All isolated rhamnolipids were tested for cytotoxicity against cancer cell lines, and compounds **1**−**3** displayed moderate activity. Additionally, compound **2,** possessing a ketone group, showed better activity than other compounds. Our research suggested that the discovery of these new rhamnolipids might be useful in expanding the field of rhamnolipid research and developing various industrial applications.

## Figures and Tables

**Table 1:** ^1^H and ^13^C NMR data for **1**−**3** in CD_3_OD.

Position	1			2			3		
	*δ*_C_	Type	*δ*_H_ (*J*, Hz)	*δ*_C_	Type	*δ*_H_ (*J*, Hz)	*δ*_C_	Type	*δ*_H_ (*J*, Hz)
3′-l-rhamnose									
1	98.9	CH	4.80, br s	98.9	CH	4.79, br s	99.0	CH	4.79, br s
2	71.2	CH	3.75, br s	71.3	CH	3.75, br s	71.5	CH	3.75, br s
3	70.9	CH	3.60, dd (9.6, 3.5)	70.9	CH	3.60, dd (9.6, 3.3)	71.2	CH	3.60, overlap
4	72.5	CH	3.35, dd (9.6, 9.6)	72.5	CH	3.35, dd (9.6, 9.6)	72.5	CH	3.35, dd (9.6, 9.6)
5	68.7	CH	3.65, dq (9.6, 6.3)	68.7	CH	3.65, dq (9.6, 6.2)	68.8	CH	3.65, overlap
6	16.2	CH_3_	1.23, d (6.3)	16.4	CH_3_	1.23, d (6.2)	16.5	CH_3_	1.23, d (6.2)
1′	173.9	C		174.6	C		174.0	C	
2′	40.0	CH_2_	2.53, dd (13.7, 7.5)	40.5	CH_2_	2.52, dd (14.8, 7.2)	40.0	CH_2_	2.52, dd (14.4, 7.6)
			2.48, dd (13.7, 5.1)			2.47, dd (14.8, 5.4)			2.48, dd (14.4, 5.1)
3′	74.2	CH	4.08, m	74.4	CH	4.08, m	74.2	CH	4.08, m
4′	33.1	CH_2_	1.55, m	33.1	CH_2_	1.56, m	33.1	CH_2_	1.32, 1.57, m
5′	24.5	CH_2_	1.29, m	24.5	CH_2_	1.30, m	24.5	CH_2_	1.32, 1.58, m
6′–15′	24.5–29.4	CH_2_	1.29–1.45, overlap	24.5–29.4	CH_2_	1.29–1.45, overlap	24.5–29.4	CH_2_	1.29–1.45, overlap
16′	38.8	CH_2_	1.39, m	42.9	CH_2_	2.47, t (7.4)	36.9	CH_2_	1.45, 1.53, m
17′	67.1	CH	3.70, m	210.8	C		70.9	CH	3.70, m
18′	22.0	CH_3_	1.13, d (6.2)	28.3	CH_3_	2.12, s	17.8	CH_3_	1.12, d (6.0)
2-l-rhamnose									
1′′							97.5	CH	4.79, br s
2′′							71.4	CH	3.72, br s
3′′							71.1	CH	3.60, overlap
4′′							72.5	CH	3.33, dd (9.6, 9.6)
5′′							68.6	CH	3.65, overlap
6′′							16.4	CH_3_	1.23, d (6.2)

The assignments were aided by ^1^H–^1^H COSY, ROESY, HSQC, and HMBC NMR spectra.

**Table 2:** Growth inhibition (GI_50_, μM) values of **1**–**3** against human tumor cell lines.

Cell Lines ^a^	GI_50_ ± SD (μM)			
	1	2	3	ADR ^b^
HCT-15	41.5 ± 4.3	16.7 ± 0.4	26.9 ± 2.0	0.173 ± 0.007
NUGC-3	30.6 ± 3.6	19.3 ± 1.9	32.0 ± 4.7	0.119 ± 0.009
NCI-H23	27.5 ± 3.3	13.7 ± 0.7	36.9 ± 4.7	0.130 ± 0.009
ACHN	34.2 ± 1.7	14.1 ± 0.3	29.1 ± 1.0	0.163 ± 0.012
PC-3	37.8 ± 0.1	18.2 ± 3.0	33.3 ± 3.3	0.145 ± 0.001
MDA-MB-231	30.6 ± 0.4	40.4 ± 1.3	25.5 ± 1.6	0.128 ± 0.011

^a^ HCT-15: Colon cancer, NUGC-3: Stomach cancer, NCI-H23: Lung cancer, ACHN: Renal cancer, PC-3: Prostate cancer, MDA-MB-231: Breast cancer; GI_50_ values are the concentration corresponding to 50% growth inhibition. ^b^ ADR: Adriamycin as standard. SD: Standard deviation.

## Supplementary Materials

The following are available online at https://www.mdpi.com/1660-3397/17/4/237/s1, Figures S1–S10: HRESI-MS data, ^1^H NMR, ^13^C NMR, COSY, HSQC, HMBC, ROESY and experimental spectra of **1**; Figures S11–S26: HRESI-MS data, ^1^H NMR, ^13^C NMR, COSY, HSQC, HMBC and experimental spectra of **2** and **3**.

## References

[1] Chong H., Li Q. (2017). Microbial Production of Rhamnolipids: Opportunities, Challenges and Strategies. Microb. Cell Fact..

[2] Rikalovic M., Gojgic-Cvijovic G.M., Vrvic M., Karadzic I. (2012). Production and Characterization of Rhamnolipids from Pseudomonas Aeruginosa San Ai. J. Serb. Chem. Soc..

[3] Rashedi H., Mazaheri Assadi M., Bonakdarpour B., Jamshidi E. (2005). Environmental Importance of Rhamnolipid Production from Molasses as a Carbon Source. Int. J. Environ. Sci. Technol..

[4] Chayabutra C., Wu J., Ju L.K. (2001). Rhamnolipid Production by Pseudomonas Aeruginosa Under Denitrification: Effects of Limiting Nutrients and Carbon Substrates. Biotechnol. Bioeng..

[5] Gunther N.W., Nunez A., Fett W., Solaiman D.K. (2005). Production of Rhamnolipids by Pseudomonas Chlororaphis, a Nonpathogenic Bacterium. Appl. Environ. Microbiol..

[6] Ławniczak Ł., Marecik R., Chrzanowski Ł. (2013). Contributions of Biosurfactants to Natural or Induced Bioremediation. Appl. Microbiol. Biotechnol..

[7] Hoskova M., Schreiberova O., Jezdik R., Chudoba J., Masak J., Sigler K., Rezanka T. (2013). Characterization of Rhamnolipids Produced by Non-Pathogenic Acinetobacter and Enterobacter Bacteria. Bioresour. Technol..

[8] Sinumvayo J.P. (2015). Agriculture and Food Applications of Rhamnolipids and its Production by Pseudomonas Aeruginosa. J. Chem. Eng. Process Technol..

[9] Soares dos Santos A., Pereira N., Freire D.M.G. (2016). Strategies for Improved Rhamnolipid Production by Pseudomonas Aeruginosa PA1. PeerJ.

[10] Lang S., Wullbrandt D. (1999). Rhamnose lipids–Biosynthesis, Microbial Production and Application Potential. Appl. Microbiol. Biotechnol..

[11] Mulligan C., Mulligan C.N. (2005). Environmental Applications for Biosurfactants. Environ. Pollut..

[12] Sekhon Randhawa K.K., Rahman P.K.S.M. (2014). Rhamnolipid Biosurfactants—Past, Present, and Future Scenario of Global Market. Front. Microbiol..

[13] Trischman J.A., Jensen P.R., Fenical W. (1994). Halobacillin: A Cytotoxic Cyclic Acylpeptide of the Iturin Class Produced by a Marine Bacillus. Tetrahedron Lett..

[14] Xiong Z.Q., Wang J.F., Hao Y.Y., Wang Y. (2013). Recent Advances in the Discovery and Development of Marine microbial Natural Products. Mar. Drugs.

[15] Javed F., Qadir M.I., Janbaz K.H., Ali M. (2011). Novel Drugs from Marine Microorganisms. Crit. Rev. Microbiol..

[16] Song S.J., Park J., Ryu J., Rho H.S., Kim W., Kim J.S. (2017). Biodiversity Hotspot for Marine Invertebrates around the Dokdo, East Sea, Korea: Ecological Checklist Revisited. Mar. Pollut. Bull..

[17] Ohtani I., Kusumi T., Kashman Y., Kakisawa H. (1991). High-Field FT NMR Application of Mosher’s Method. The absolute Configurations of Marine Terpenoids. J. Am. Chem. Soc..

[18] Shin B., Ahn S., Noh M., Shin J., Oh D.-C. (2015). Suncheonosides A–D, Benzothioate Glycosides from a Marine-Derived Streptomyces Sp.. J. Nat. Prod..

[19] Skehan P., Storeng R., Scudiero D., Monks A., McMahon J., Vistica D., Warren J.T., Bokesch H., Kenney S., Boyd M.R. (1990). New Colorimetric Cytotoxicity Assay for Anticancer-Drug Screening. J. Natl. Cancer Inst..

