# Supplementary material for: Dokdolipids A−C, Hydroxylated Rhamnolipids from the Marine-Derived Actinomycete Actinoalloteichus hymeniacidonis
Source: Mar Drugs. 2019 Apr 20;17(4):237. doi: 10.3390/md17040237 (PMC6521253; doi:10.3390/md17040237)
Supplement: Supplementary file 1 [file marinedrugs-17-00237-s001.pdf]

Supplementary data

Dokdolipids A−C, Hydroxylated Rhamnolipids from the Marine-Derived Actinomycete *Actinoalloteichus hymeniacidonis*

Byeoung-Kyu Choi[1,2], Hwa-Sun Lee[2], Jong Soon Kang[3] and Hee Jae Shin[1,2,*]

[1] *Department of Marine Biotechnology, University of Science and Technology, 217 Gajungro Yuseong-gu, Daejeon, 34113, Korea*

[2] *Marine Natural Products Chemistry Laboratory, Korea Institute of Ocean Science and Technology, 385 Haeyang-ro, Yeongdo-gu, Busan 49111, Korea*

[3] *Laboratory Animal ResourceCenter, Korea Research Institute of Bioscience and Biotechnology, 30 Yeongudanjiro, Cheongju 28116, Korea*

Contents

Figure S1. HRESIMS data of dokdolipid A (**1**). --3

Figure S2. ^{1}H NMR spectrum of dokdolipid A (**1**). ---4

Figure S3. ^{13}C NMR spectrum of dokdolipid A (**1**). --5

Figure S4. ^{1}H-^{1}H COSY spectrum of dokdolipid A (**1**). ---6

Figure S5. HSQC spectrum of dokdolipid A (**1**). ---7

Figure S6. HMBC spectrum of dokdolipid A (**1**). ---8

Figure S7. ROESY spectrum of dokdolipid A (**1**). --9

Figure S8. ^{1}H NMR spectrum of the hydrolysate of dokdolipid A (**1**). -----------------------10

Figure S9. ^{1}H NMR spectrum of (*S*)-MTPA (**1a**) ester. ---11

Figure S10. ^{1}H NMR spectrum of (*R*)-MTPA (**1b**) ester. --12

Figure S11. HRESIMS data of dokdolipid B (**2**). --13

Figure S12. ^{1}H NMR spectrum of dokdolipid B (**2**). ---14

Figure S13. ^{13}C NMR spectrum of dokdolipid B (**2**). --15

Figure S14. ^{1}H-^{1}H COSY spectrum of dokdolipid B (**2**). ---16

Figure S15. HSQC spectrum of dokdolipid B (**2**). ---17

Figure S16. HMBC spectrum dokdolipid B (**2**). ---18

Figure S17. ^{1}H NMR spectrum of (*S*)-MTPA (**2a**) ester. ---19

Figure S18. ^{1}H NMR spectrum of (*R*)-MTPA (**2b**) ester. --20

Figure S19. HRESIMS data of dokdolipid C (**3**). --21

Figure S20. ^{1}H NMR spectrum of dokdolipid C (**3**). ---22

Figure S21. ^{13}C NMR spectrum of dokdolipid C (**3**). --23

Figure S22. ^{1}H-^{1}H COSY spectrum of dokdolipid C (**3**). ---24

Figure S23. HSQC spectrum of dokdolipid C (**3**). ---25

Figure S24. HMBC spectrum of dokdolipid C (**3**). --26

Figure S25. ^{1}H NMR spectrum of (*S*)-MTPA (**3a**) ester. ---27

Figure S26. ^{1}H NMR spectrum of (*R*)-MTPA (**3b**) ester. --28

Elemental Composition Report

Single Mass Analysis
Tolerance = 5.0 PPM / DBE: min = -1.5, max = 50.0
Element prediction: Off
Number of isotope peaks used for i-FIT = 3

Monoisotopic Mass, Even Electron Ions
82 formula(e) evaluated with 1 results within limits (all results (up to 1000) for each mass)
Elements Used:
C: 1-40 H: 1-60 O: 1-20 Na: 1-1

Minimum:				-1.5				
Maximum:		100.0	5.0	50.0				
Mass	Calc. Mass	mDa	PPM	DBE	i-FIT	Norm	Conf(%)	Formula
485.3094	485.3090	0.4	0.8	1.5	683.2	n/a	n/a	C24 H46 O8 Na

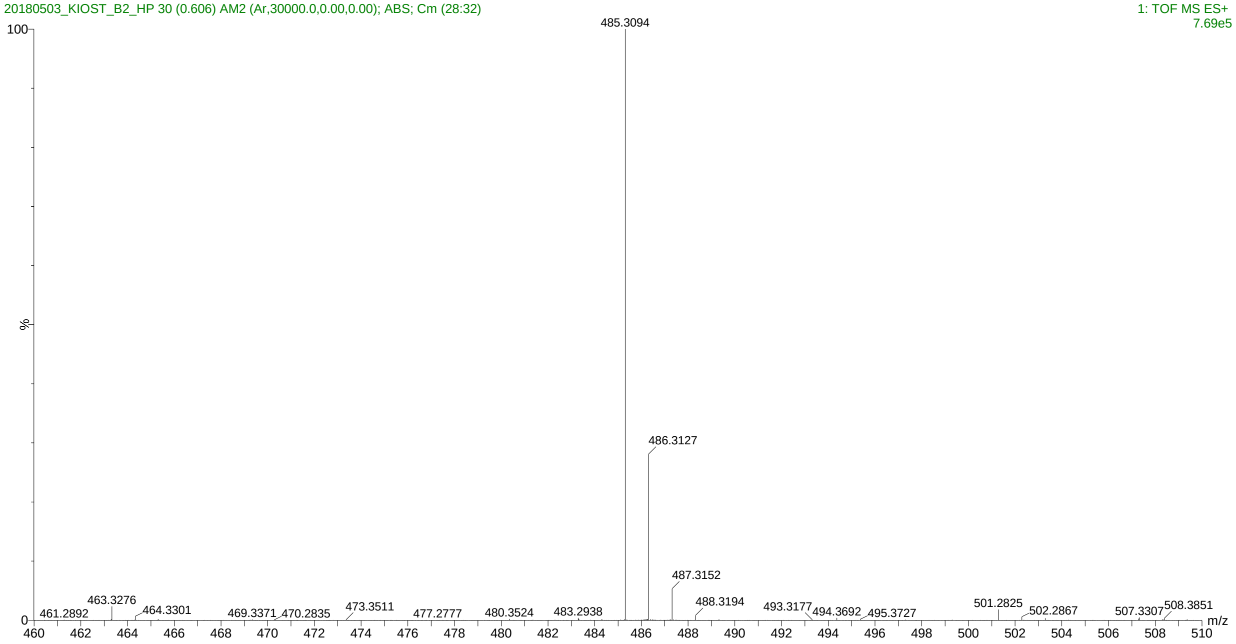

Figure S1. HRESIMS data of dokdolipid A (**1**).

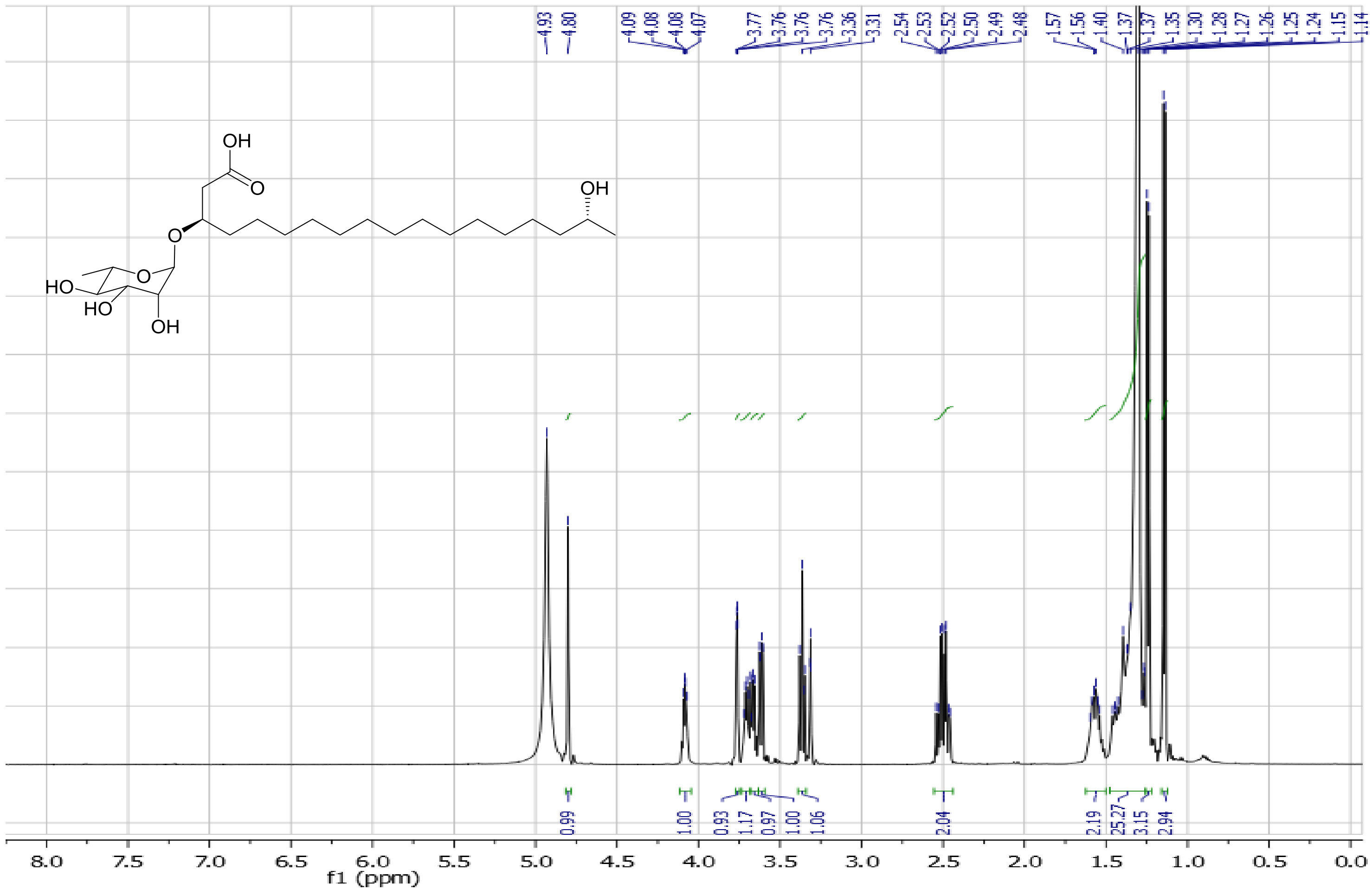

Figure S2. ^{1}H NMR spectrum of dokdolipid A (**1**).

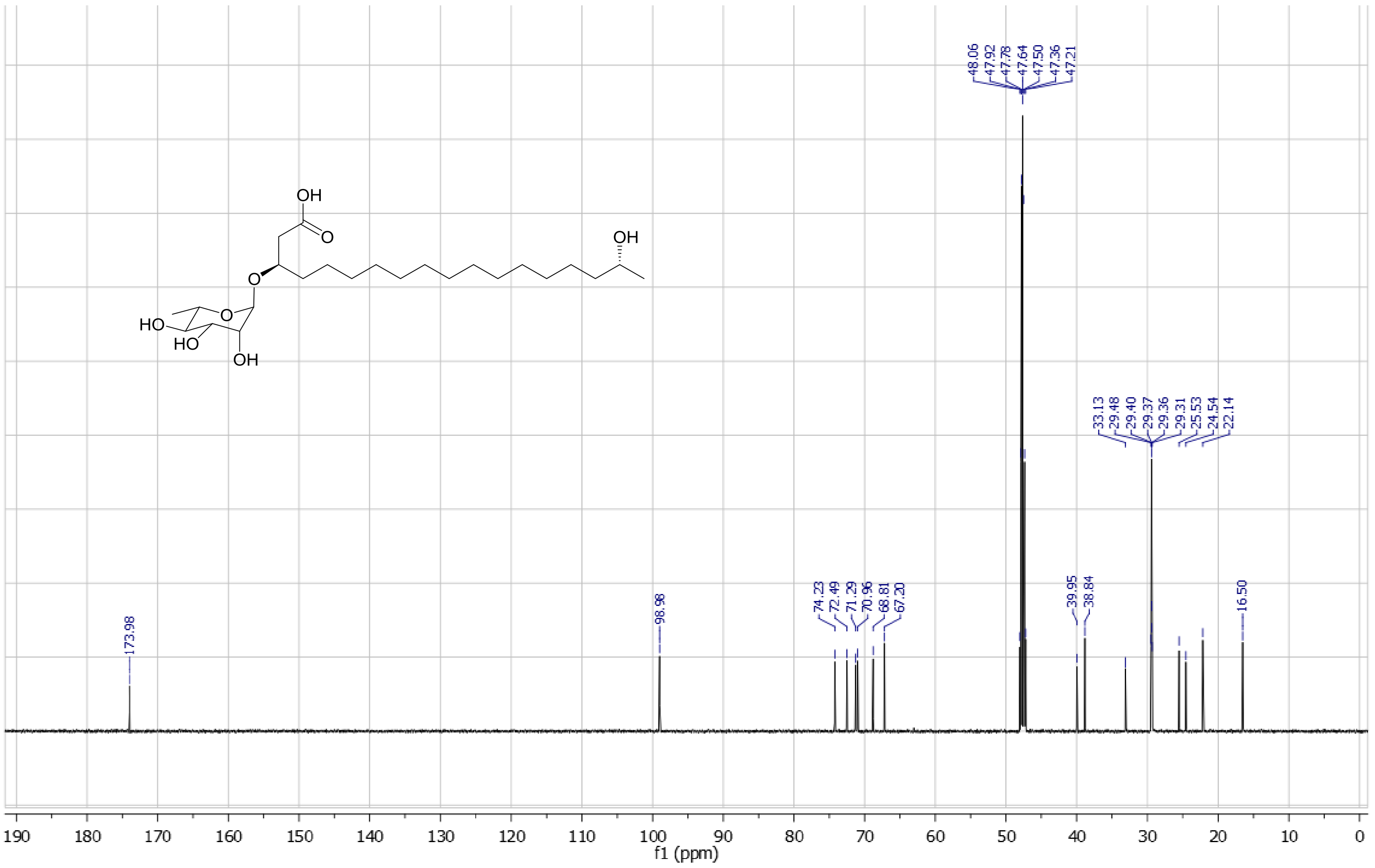

Figure S3. ^{13}C NMR spectrum of dokdolipid A (**1**).

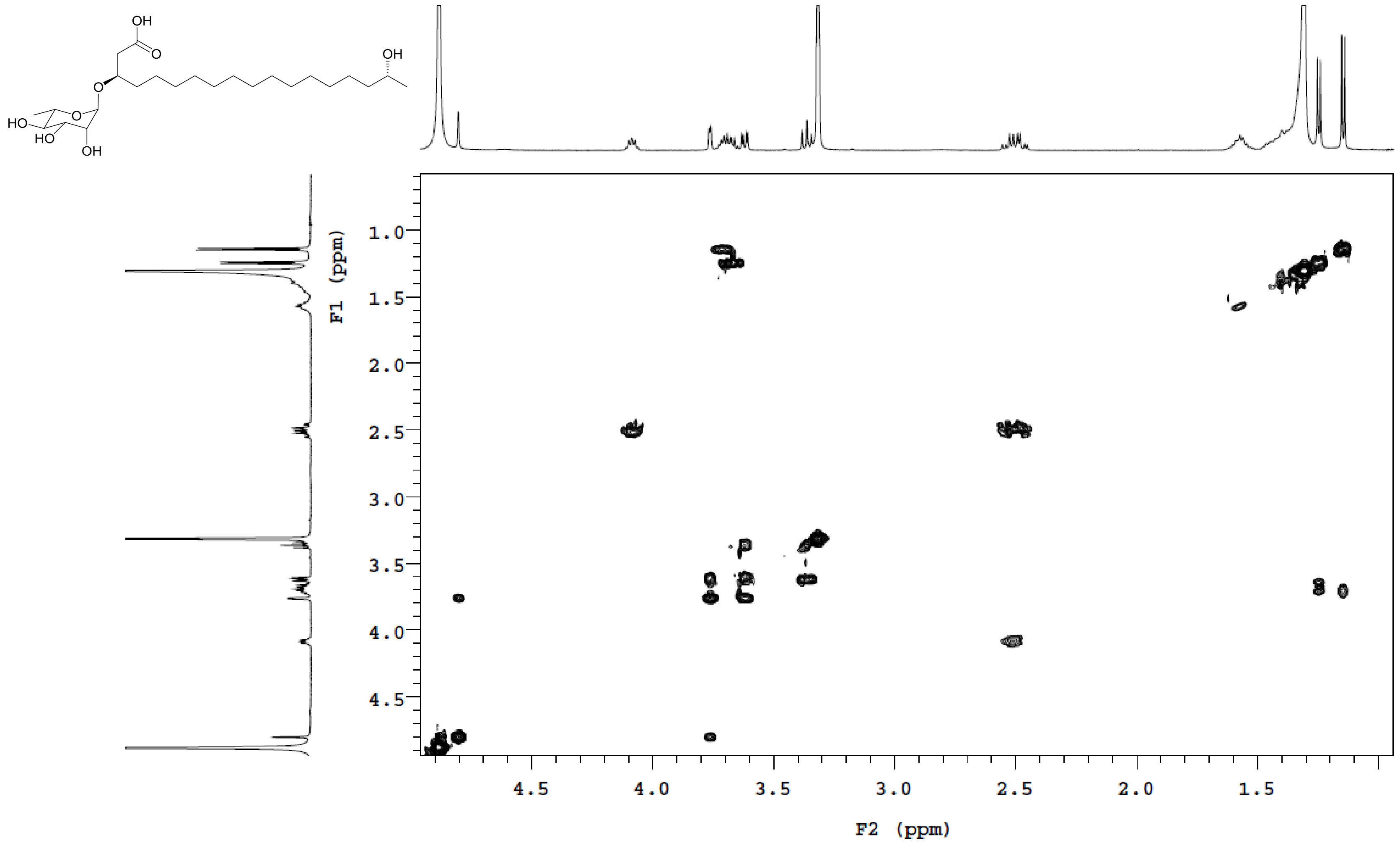

Figure S4. ^{1}H-^{1}H COSY spectrum of dokdolipid A (**1**).

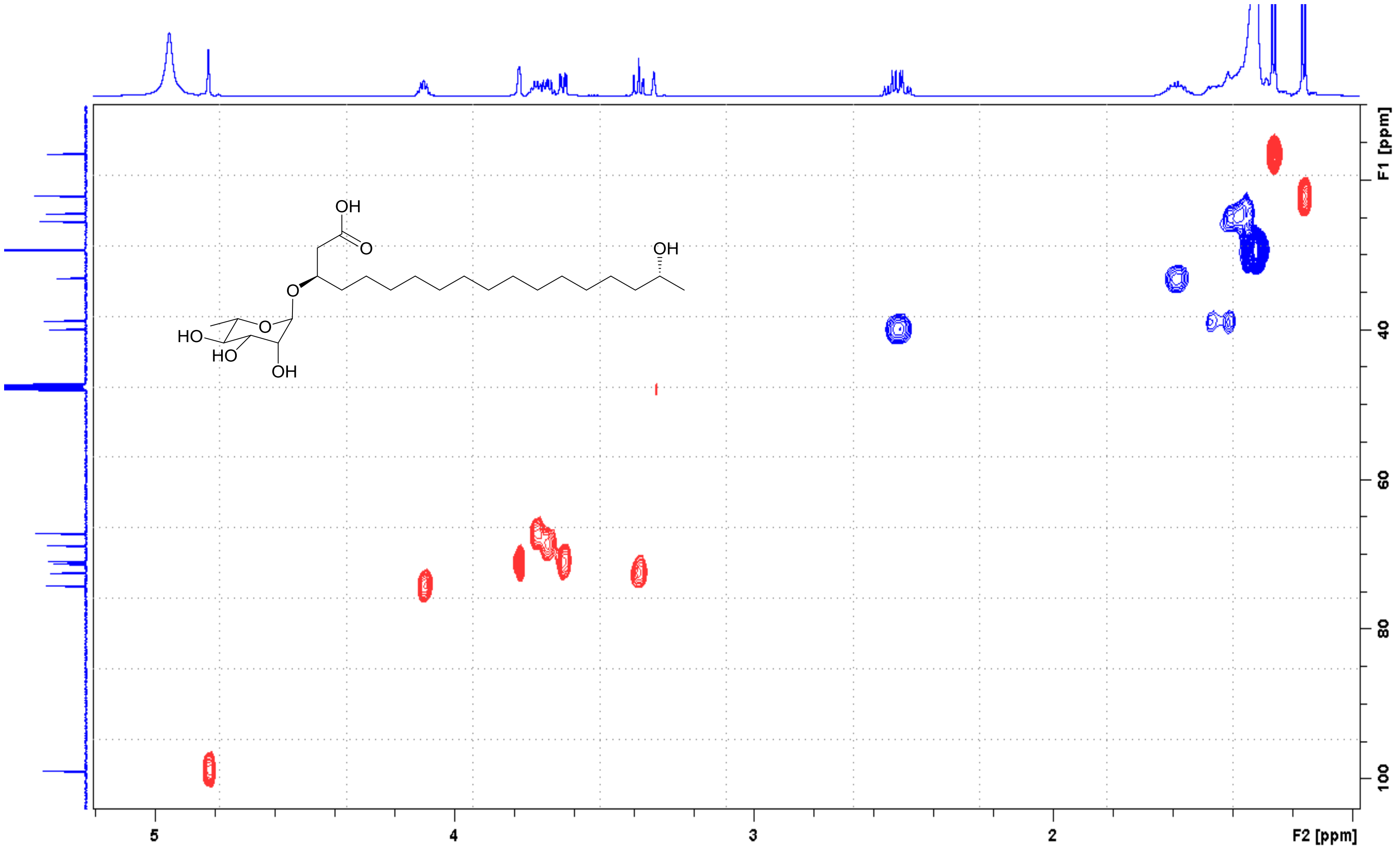

Figure S5. HSQC spectrum of dokdolipid A (**1**).

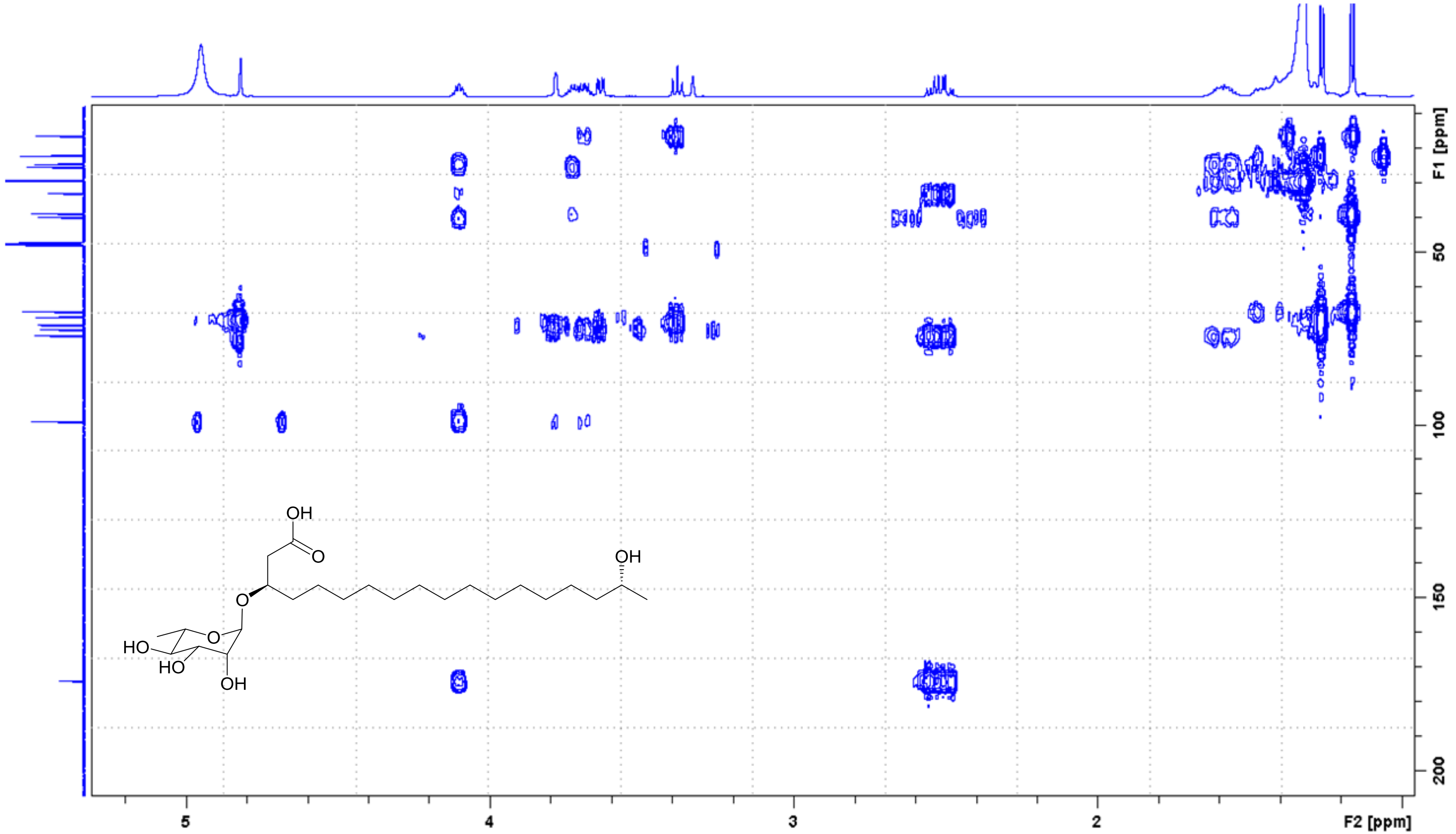

Figure S6. HMBC spectrum of dokdolipid A (**1**).

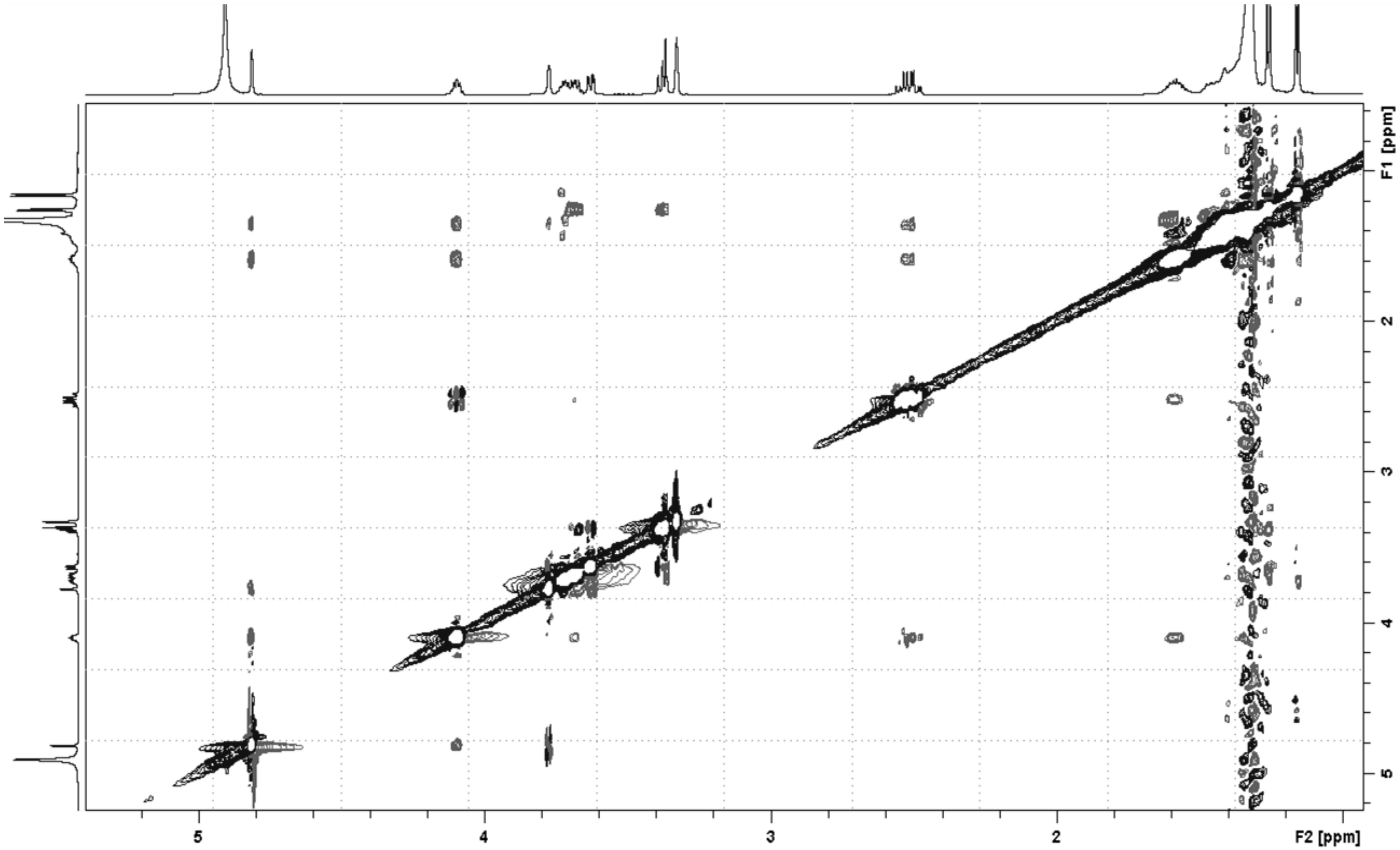

Figure S7. ROESY spectrum of dokdolipid A (**1**).

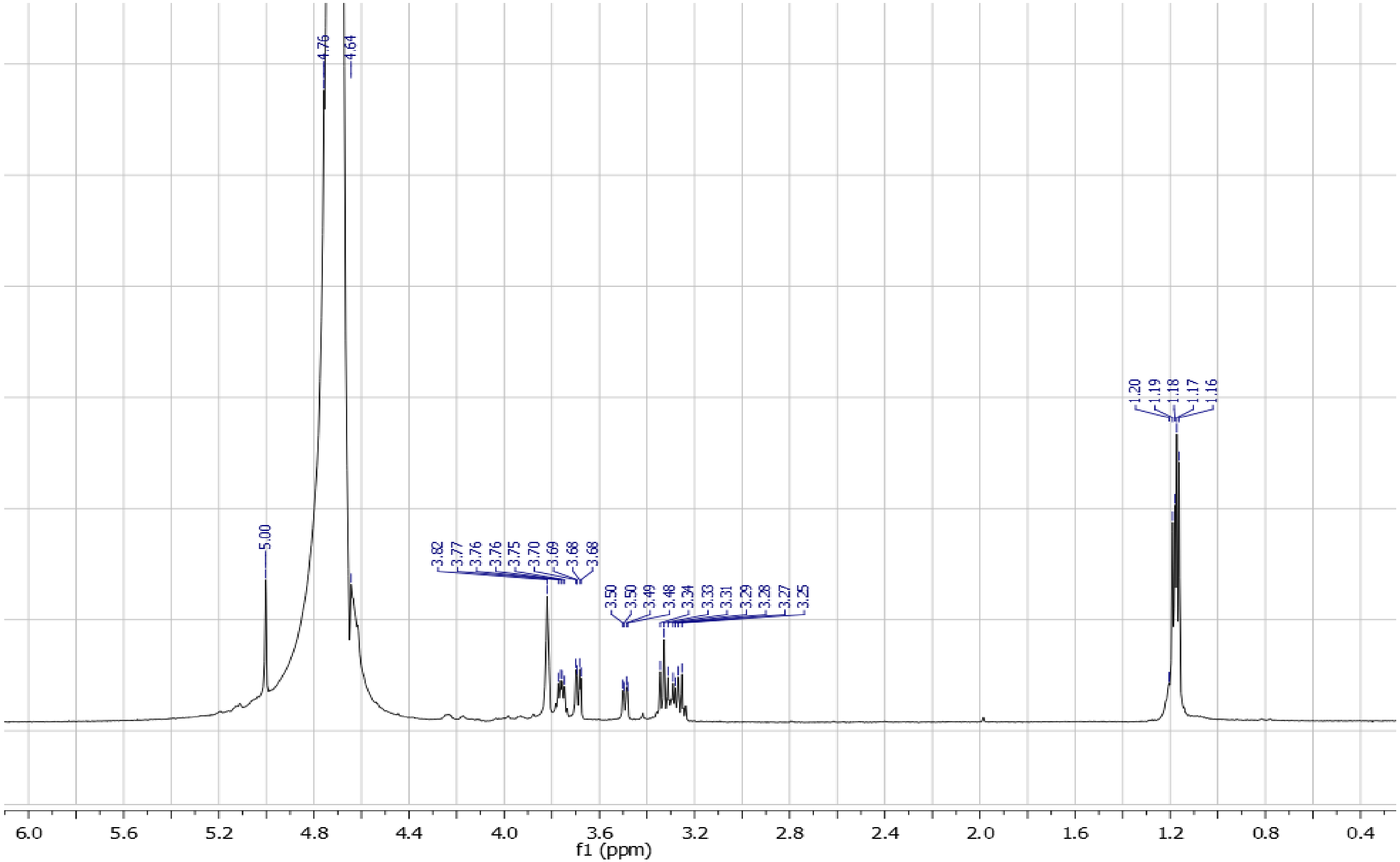

Figure S8. ^{1}H NMR spectrum of the hydrolysate of dokdolipid A (**1**).

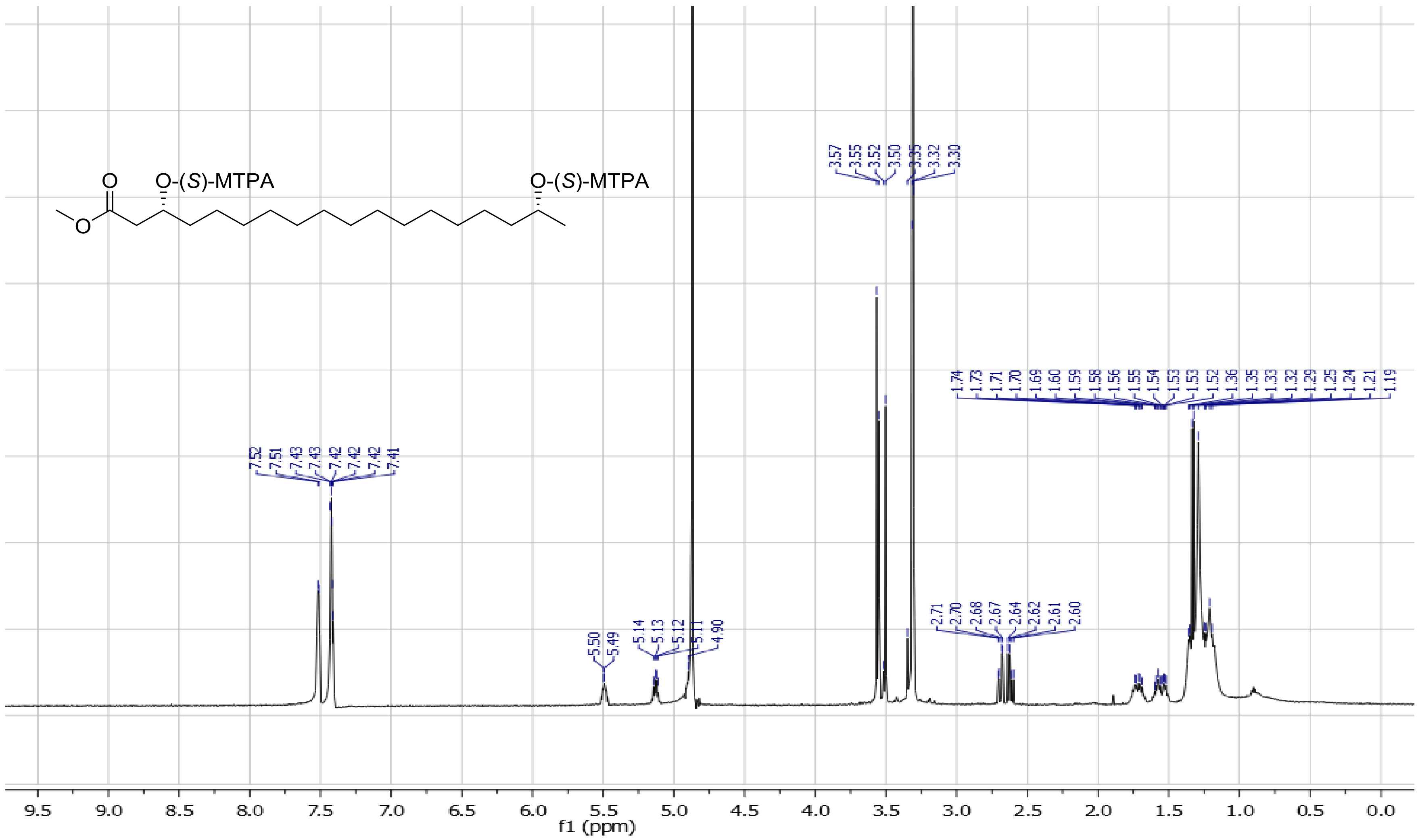

Figure S9. ^{1}H NMR spectrum of (*S*)-MTPA (**1a**) ester.

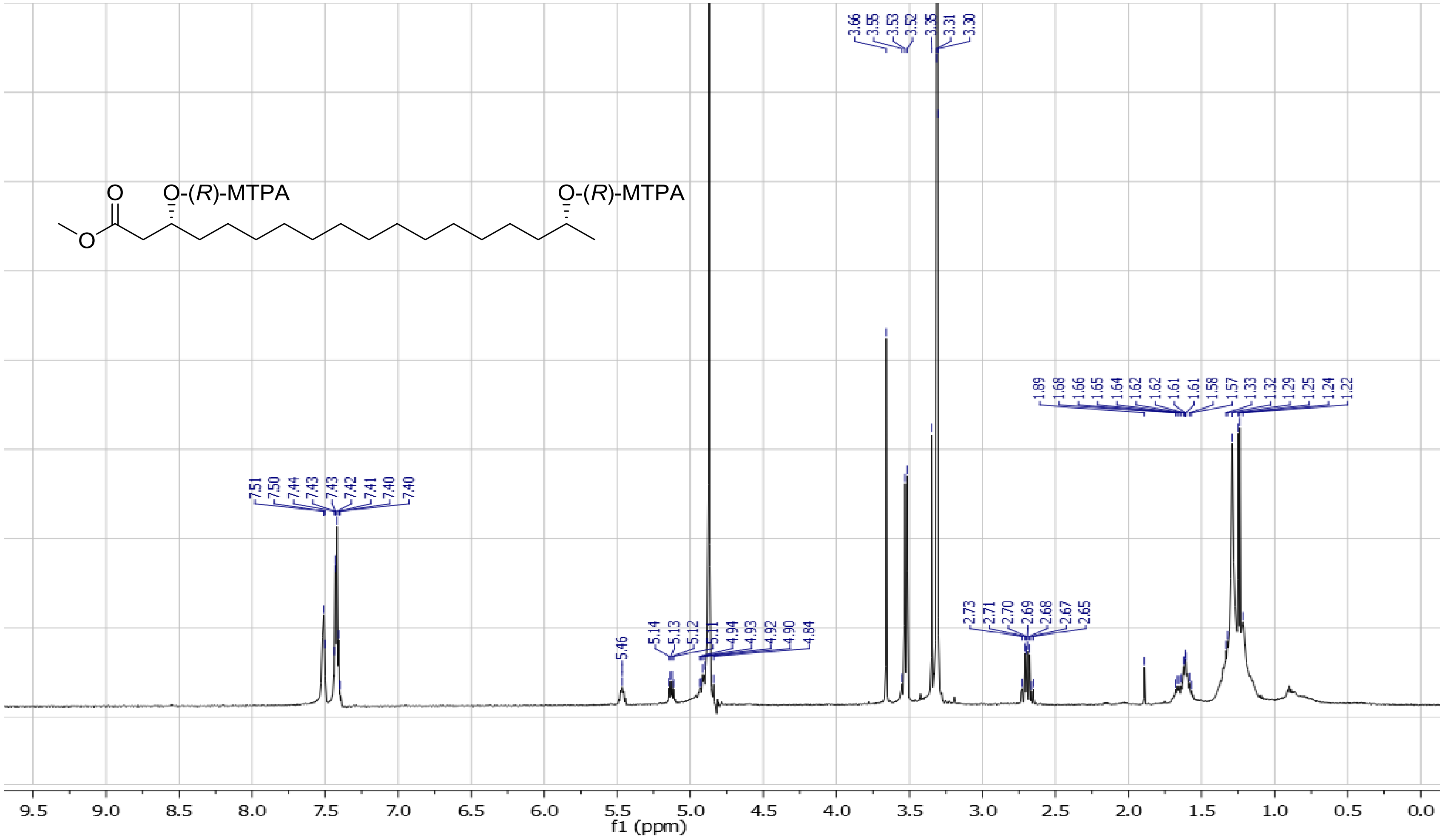

Figure S10. ^{1}H NMR spectrum of (*R*)-MTPA (**1b**) ester.

Elemental Composition Report

Single Mass Analysis
Tolerance = 5.0 PPM / DBE: min = -1.5, max = 50.0
Element prediction: Off
Number of isotope peaks used for i-FIT = 3

Monoisotopic Mass, Even Electron Ions
79 formula(e) evaluated with 1 results within limits (all results (up to 1000) for each mass)
Elements Used:
C: 1-40 H: 1-60 O: 1-20 Na: 1-1

Minimum:				-1.5				
Maximum:		100.0	5.0	50.0				
Mass	Calc. Mass	mDa	PPM	DBE	i-FIT	Norm	Conf(%)	Formula
483.2936	483.2934	0.2	0.4	2.5	686.0	n/a	n/a	C24 H44 O8 Na

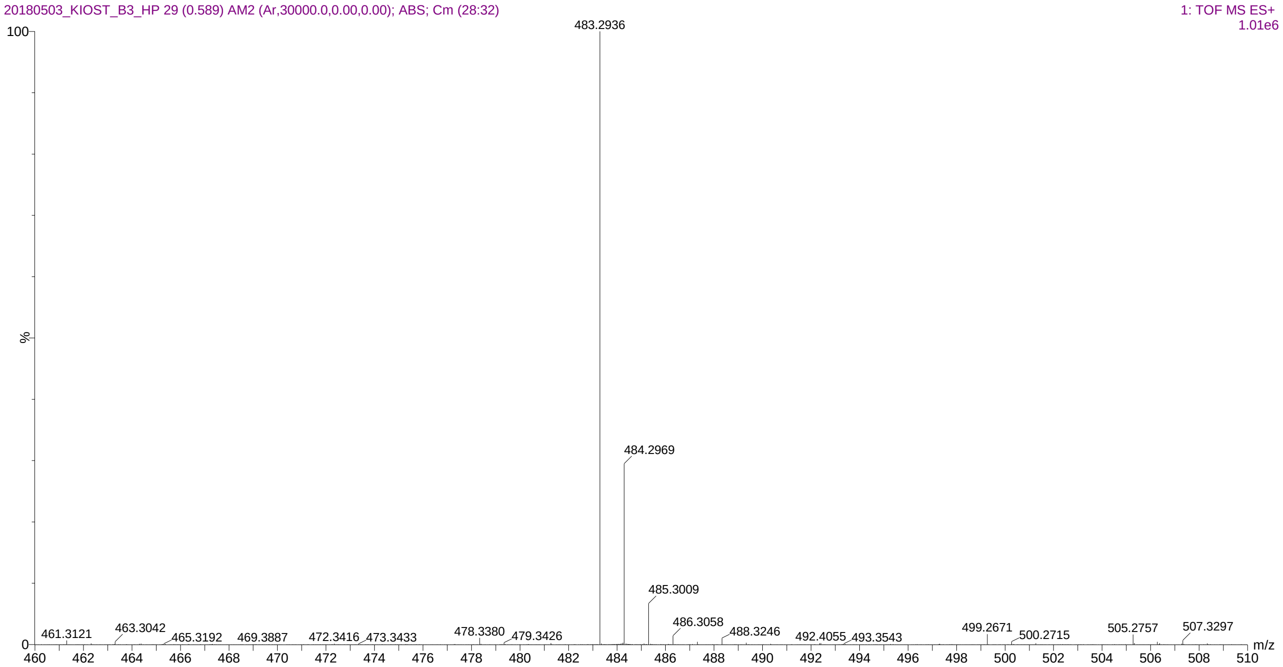

Figure S11. HRESIMS data of dokdolipid B (**2**).

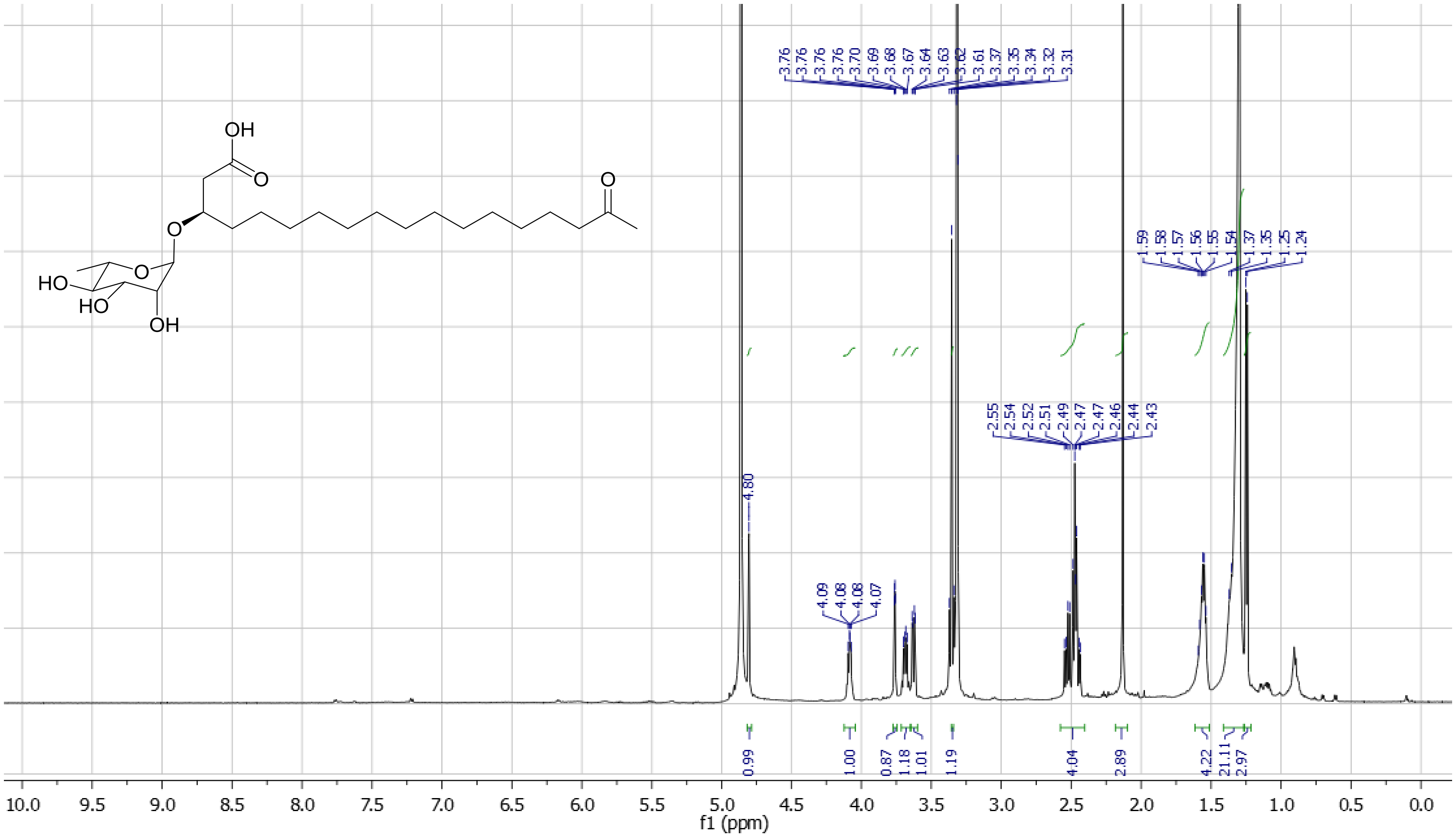

Figure S12. ^{1}H NMR spectrum of dokdolipid B (**2**).

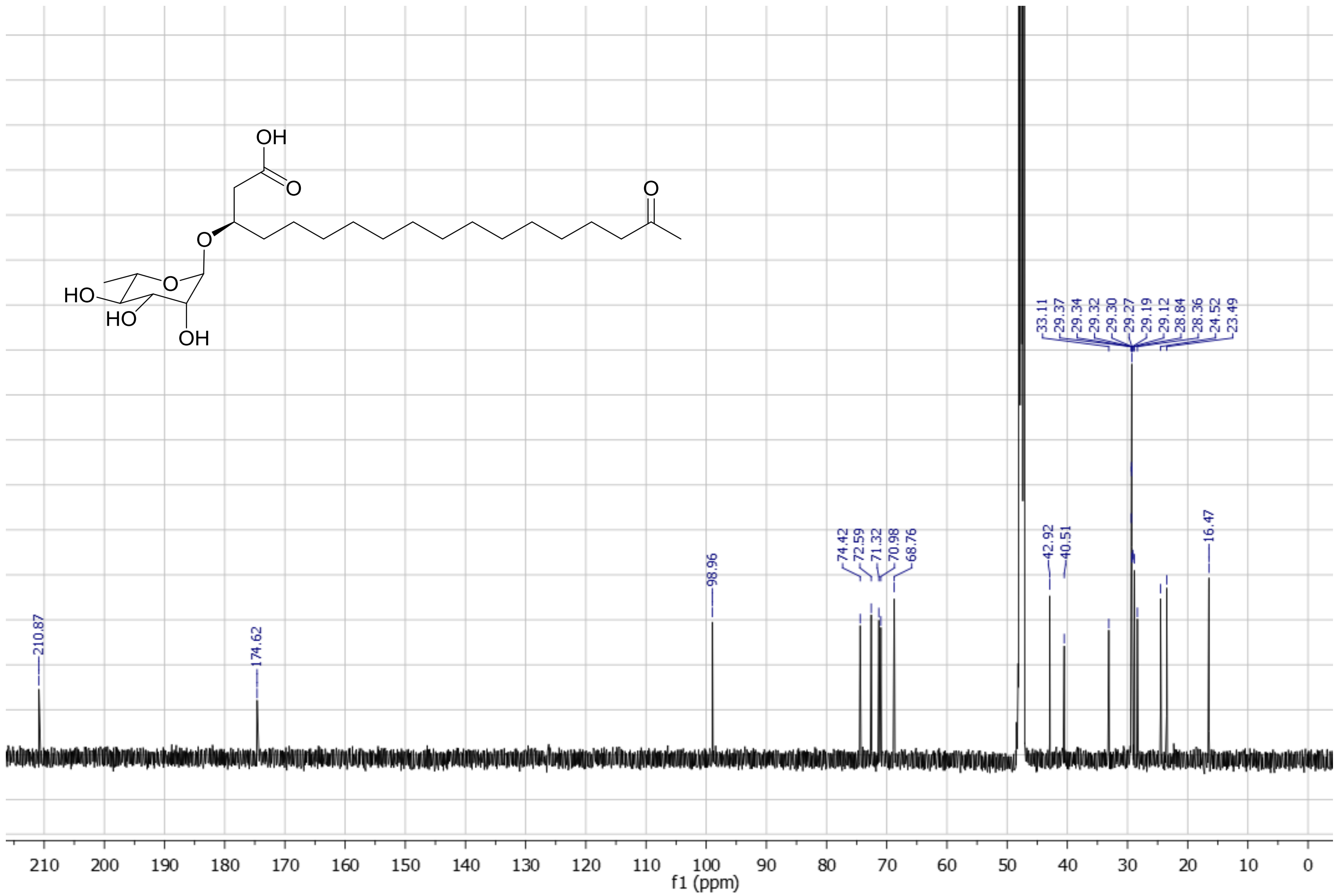

Figure S13. ^{13}C NMR spectrum of dokdolipid B (**2**).

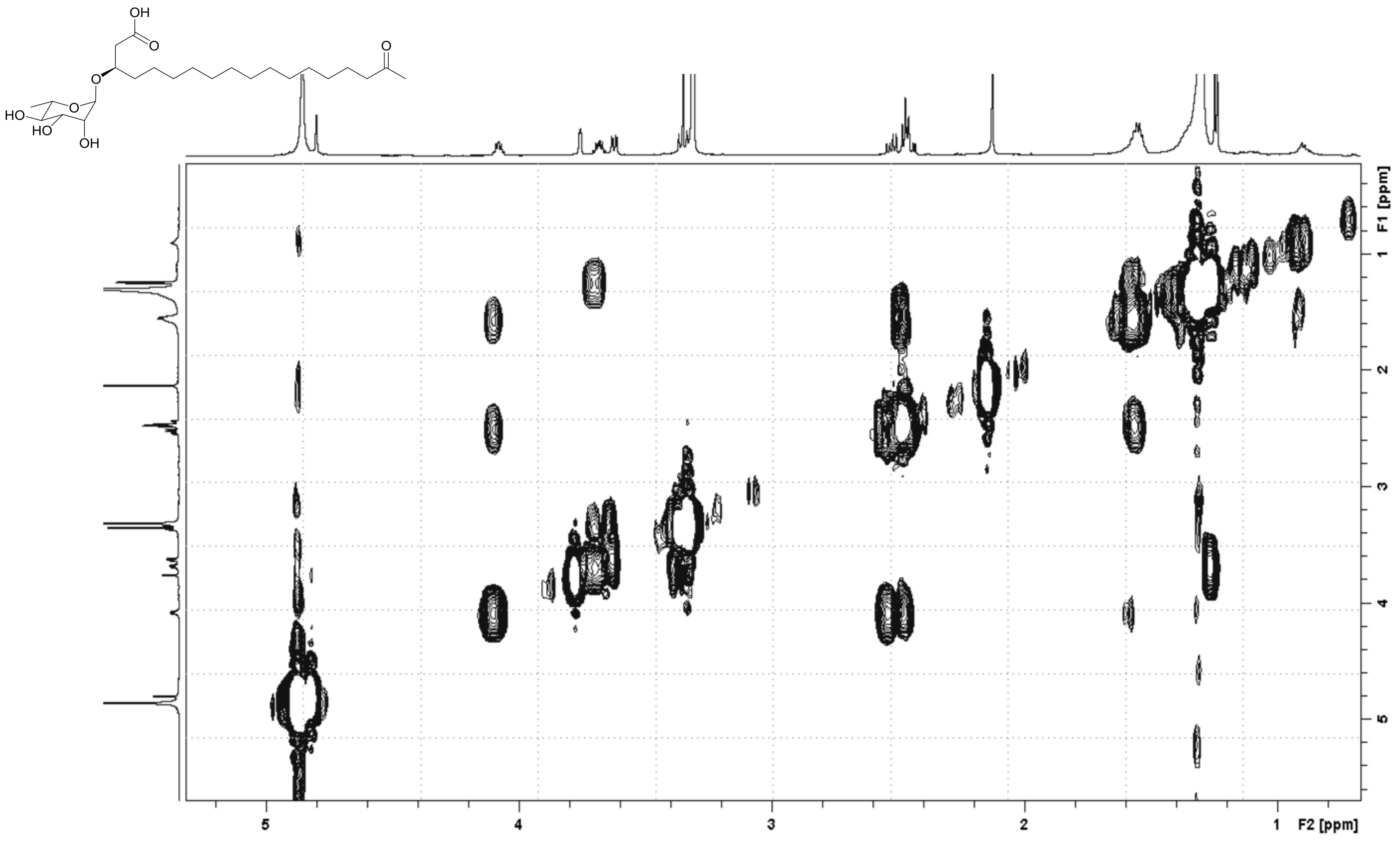

Figure S14. ^{1}H-^{1}H COSY spectrum of dokdolipid B (**2**).

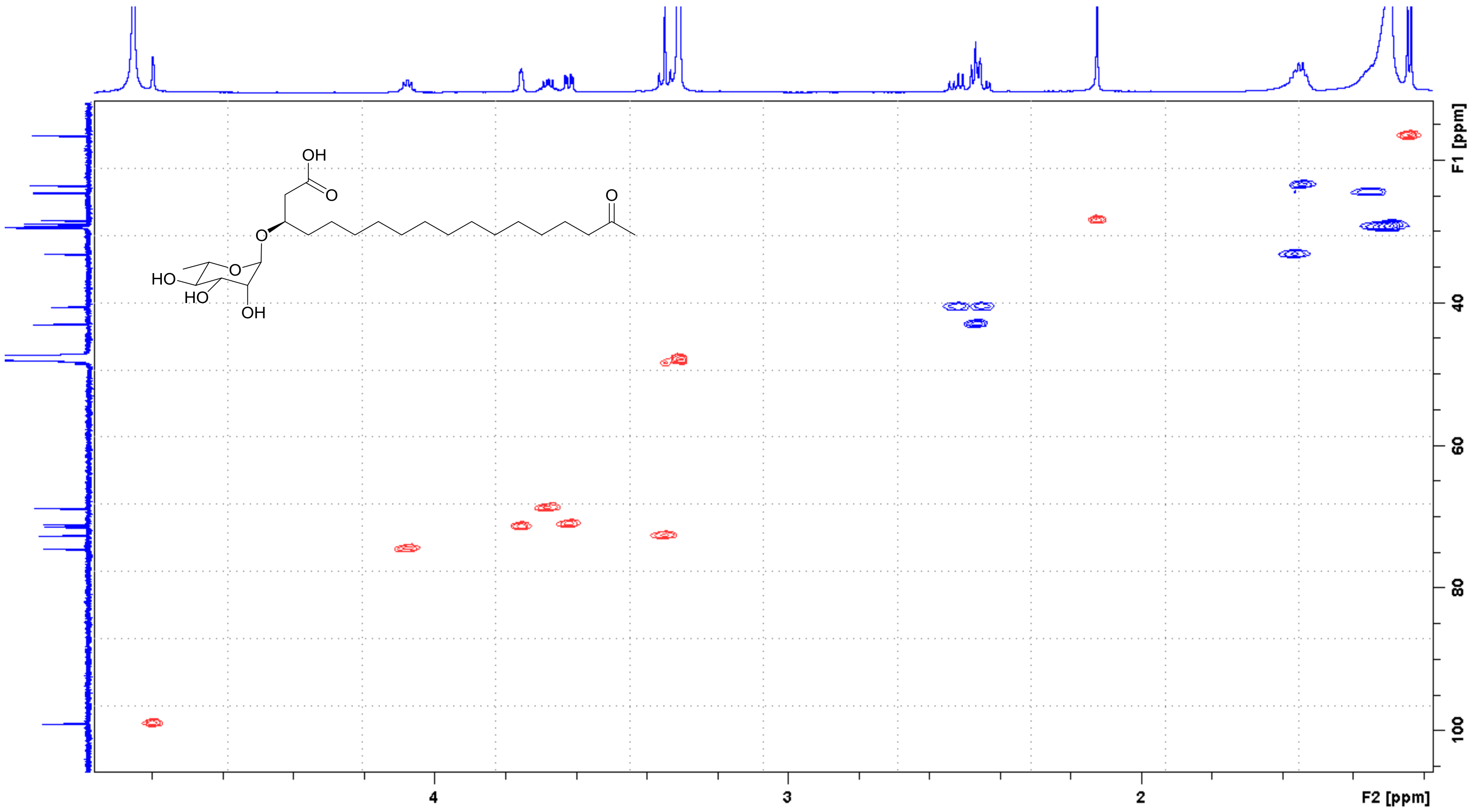

Figure S15. HSQC spectrum of dokdolipid B (**2**).

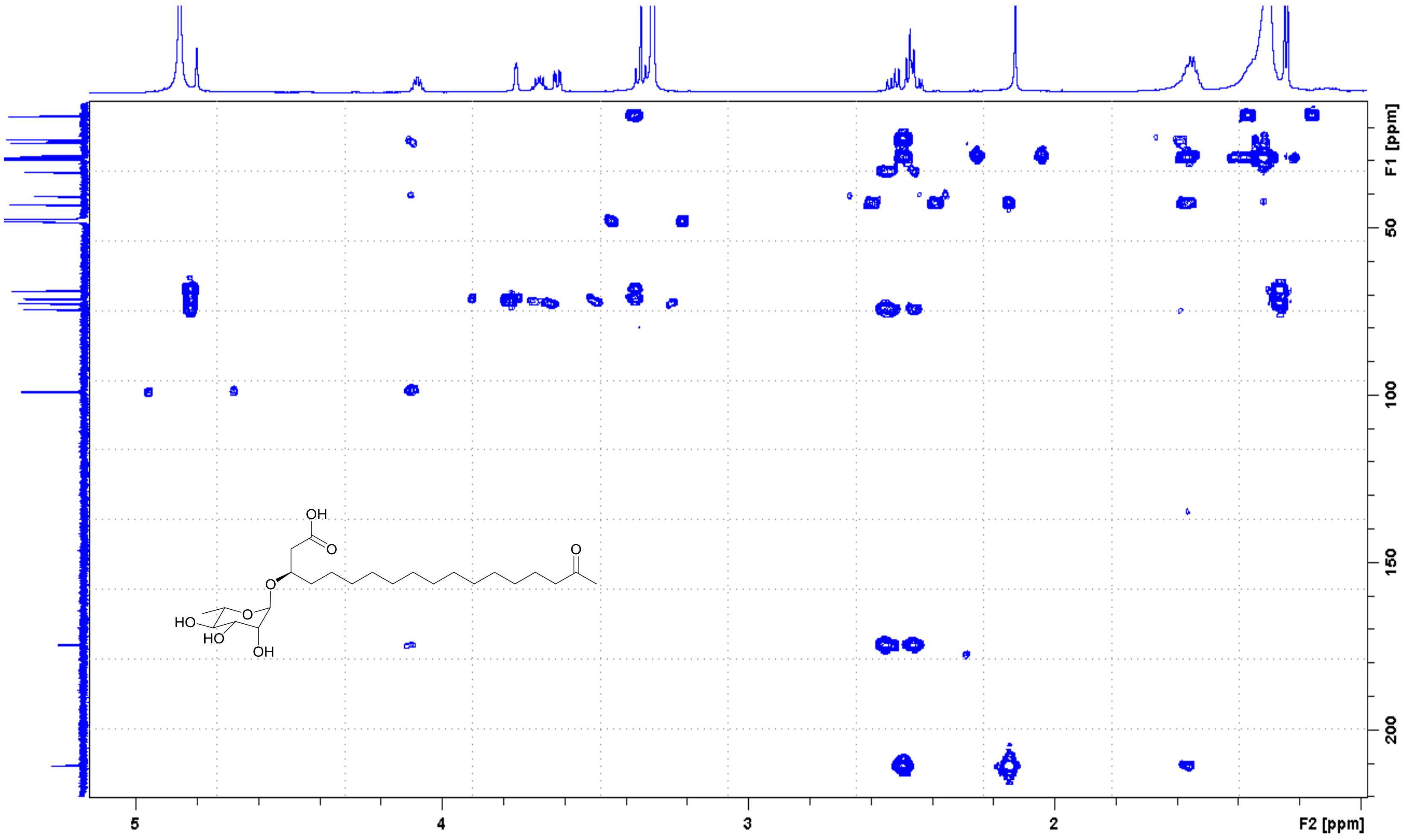

Figure S16. HMBC spectrum of dokdolipid B (**2**).

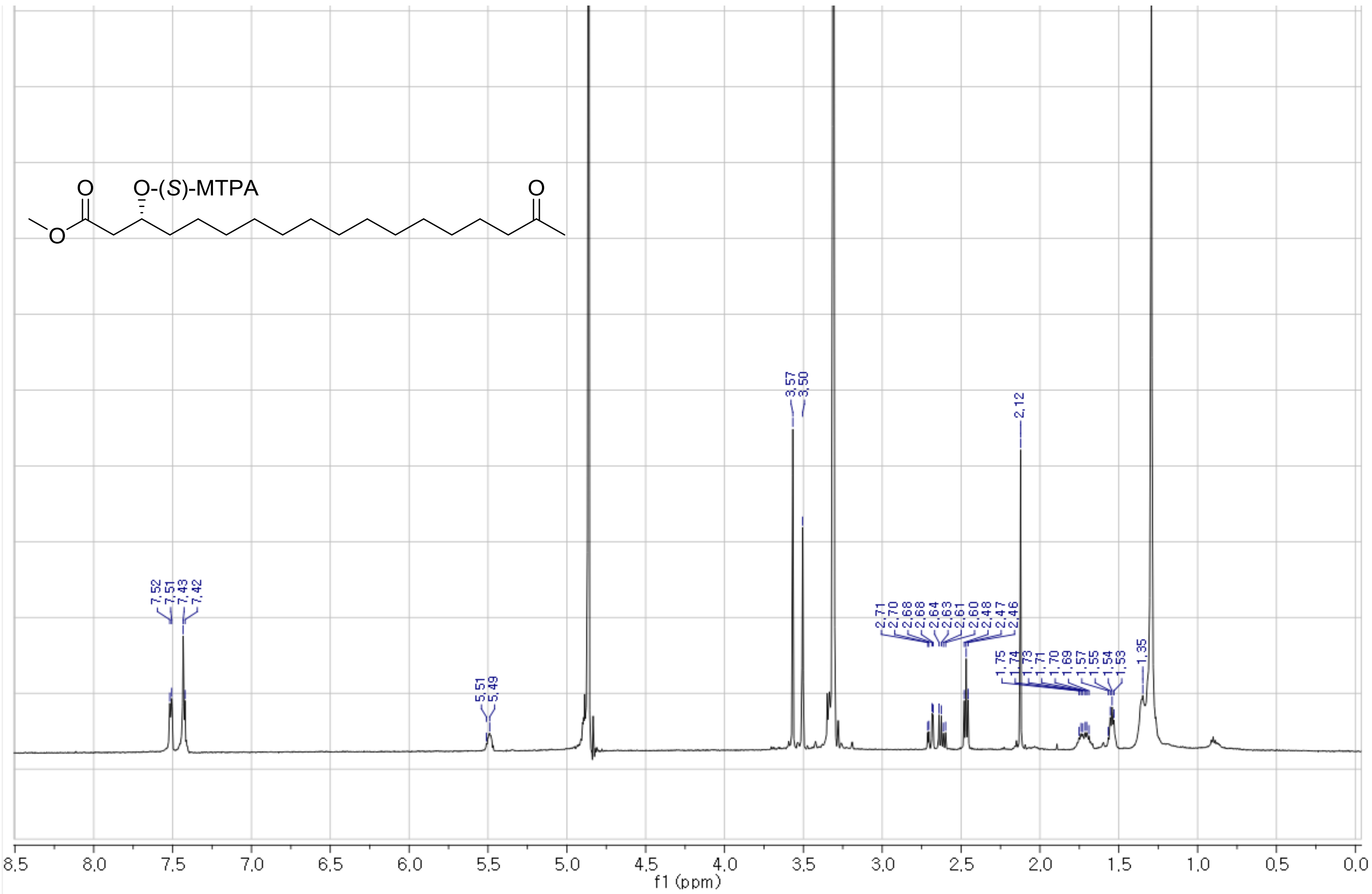

Figure S17. ^{1}H NMR spectrum of (*S*)-MTPA (**2a**) ester.

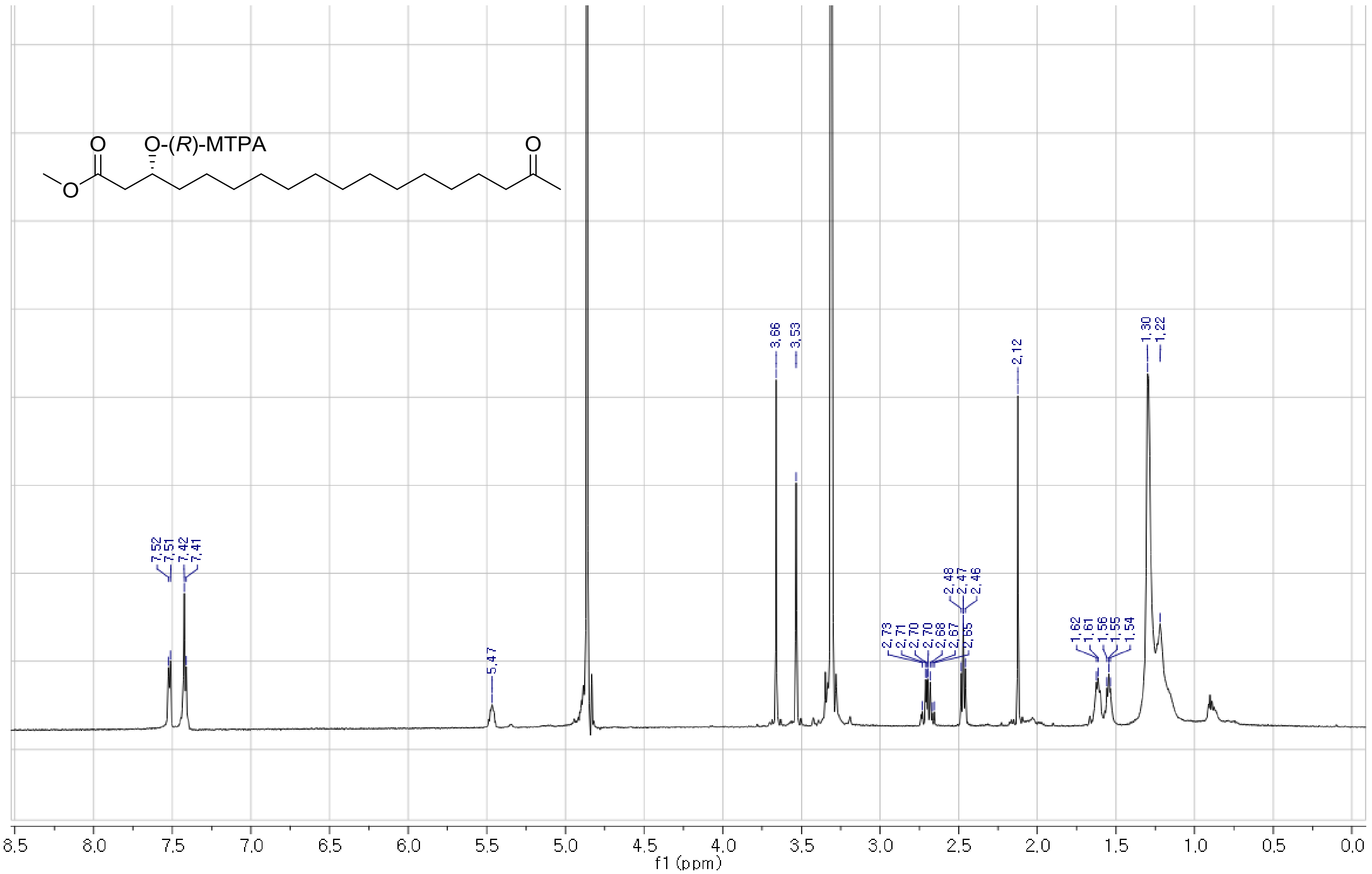

Figure S18. ^{1}H NMR spectrum of (*R*)-MTPA (**2b**) ester.

Elemental Composition Report

Single Mass Analysis
Tolerance = 5.0 PPM / DBE: min = -1.5, max = 50.0
Element prediction: Off
Number of isotope peaks used for i-FIT = 3

Monoisotopic Mass, Even Electron Ions
73 formula(e) evaluated with 1 results within limits (all results (up to 1000) for each mass)
Elements Used:
C: 1-40 H: 1-60 O: 1-20 Na: 1-1

Minimum:				-1.5				
Maximum:		100.0	5.0	50.0				
Mass	Calc. Mass	mDa	PPM	DBE	i-FIT	Norm	Conf(%)	Formula
631.3669	631.3669	0.0	0.0	2.5	694.0	n/a	n/a	C30 H56 O12 Na

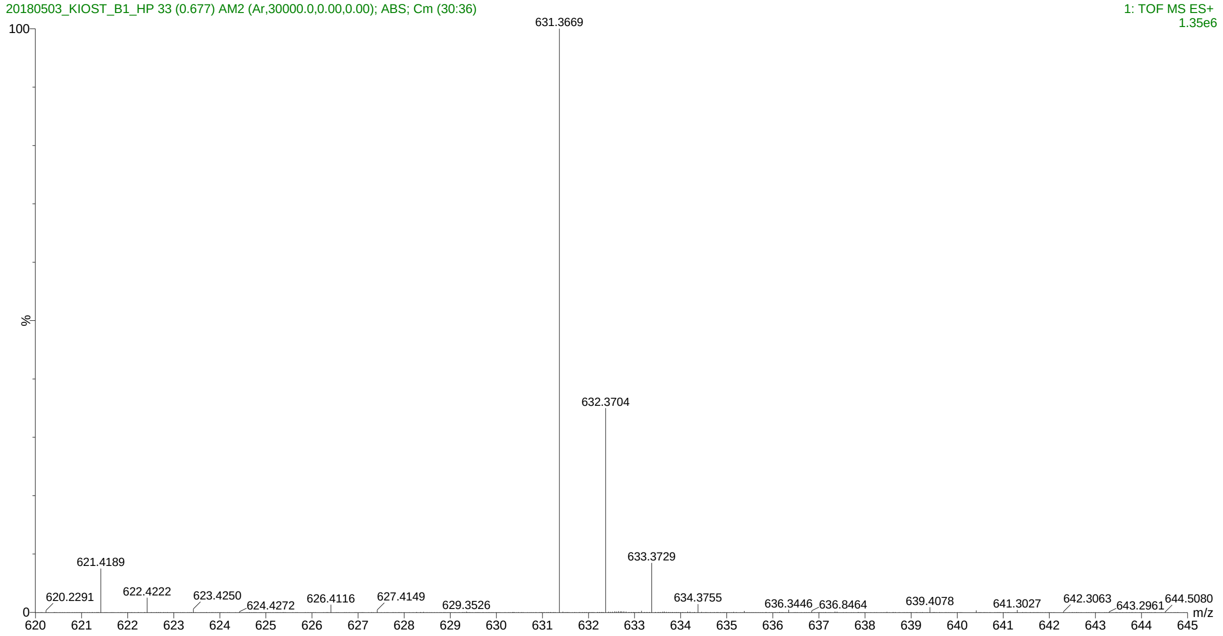

Figure S19. HRESIMS data of dokdolipid C (**3**).

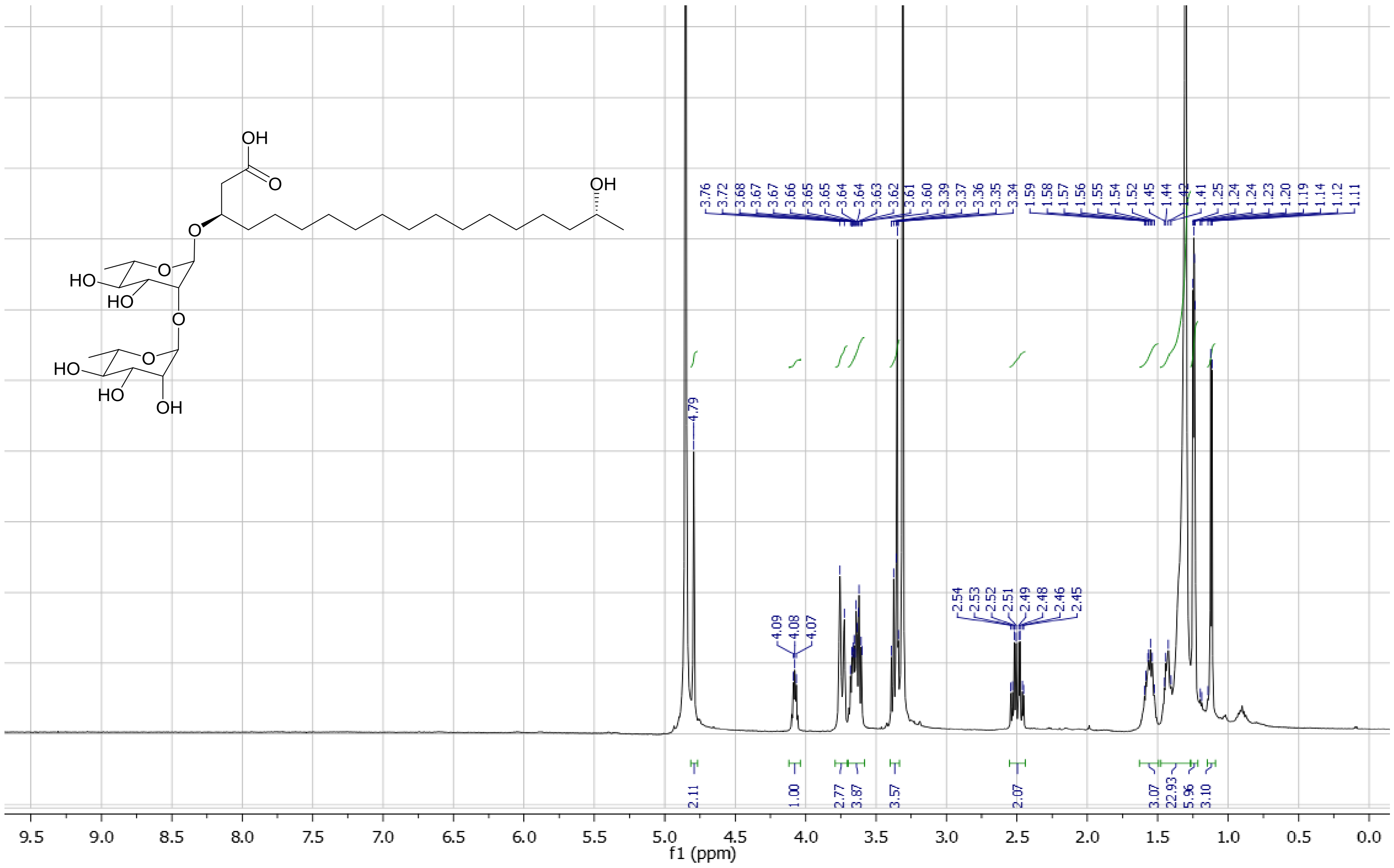

Figure S20. ^{1}H NMR spectrum of dokdolipid C (**3**).

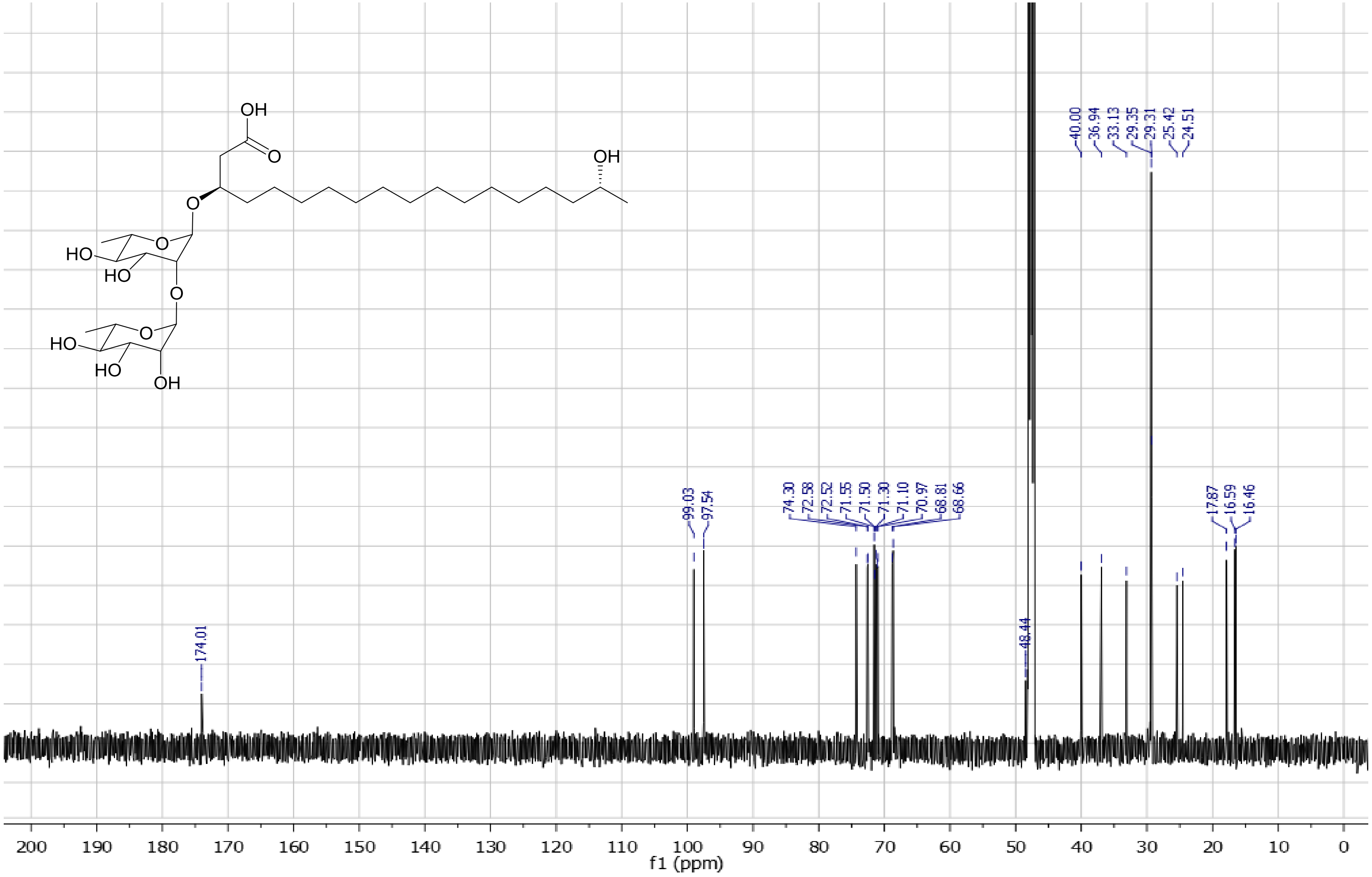

Figure S21. ^{13}C NMR spectrum of dokdolipid C (**3**).

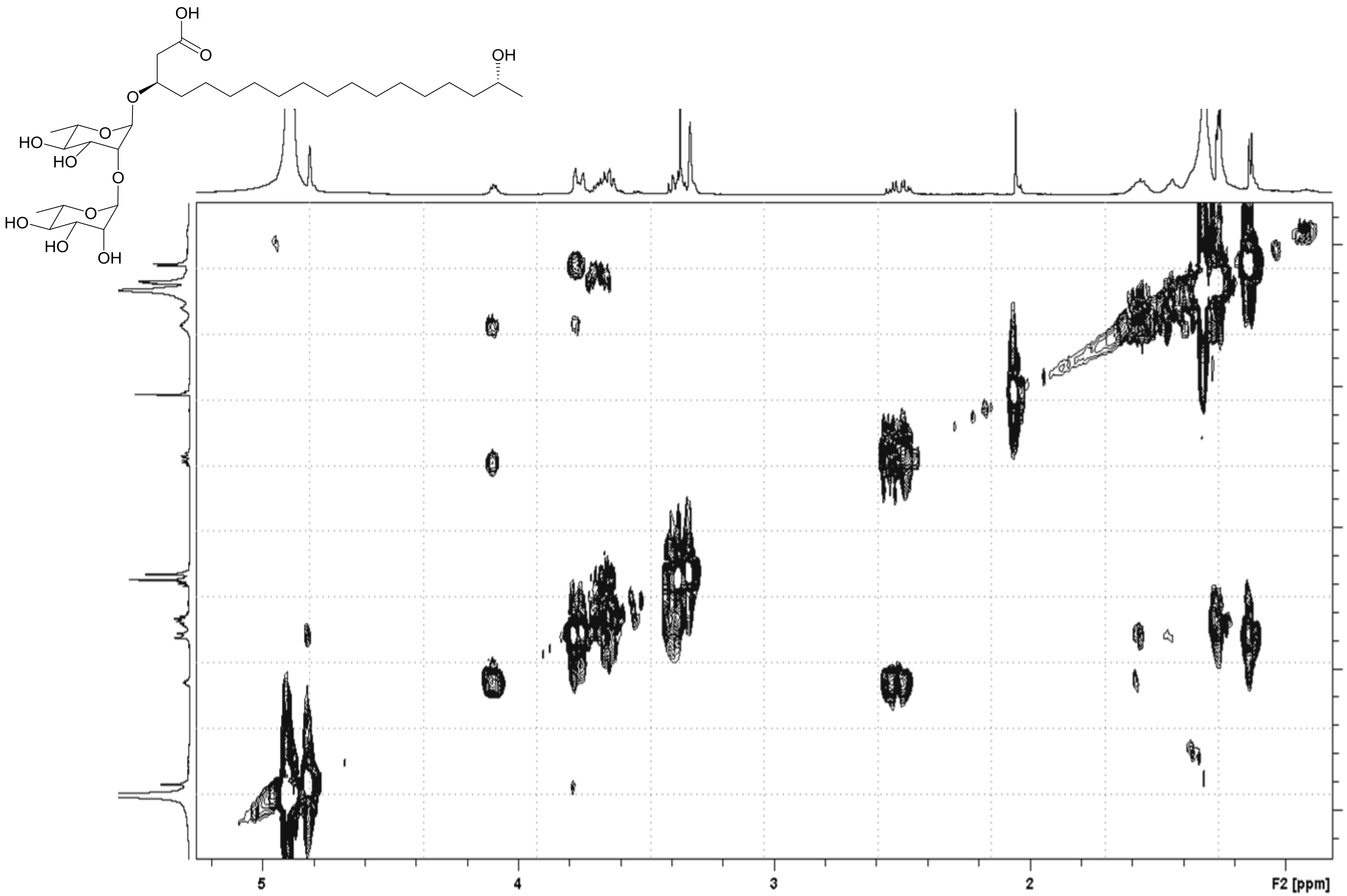

Figure S22. ^{1}H-^{1}H COSY spectrum of dokdolipid C (**3**).

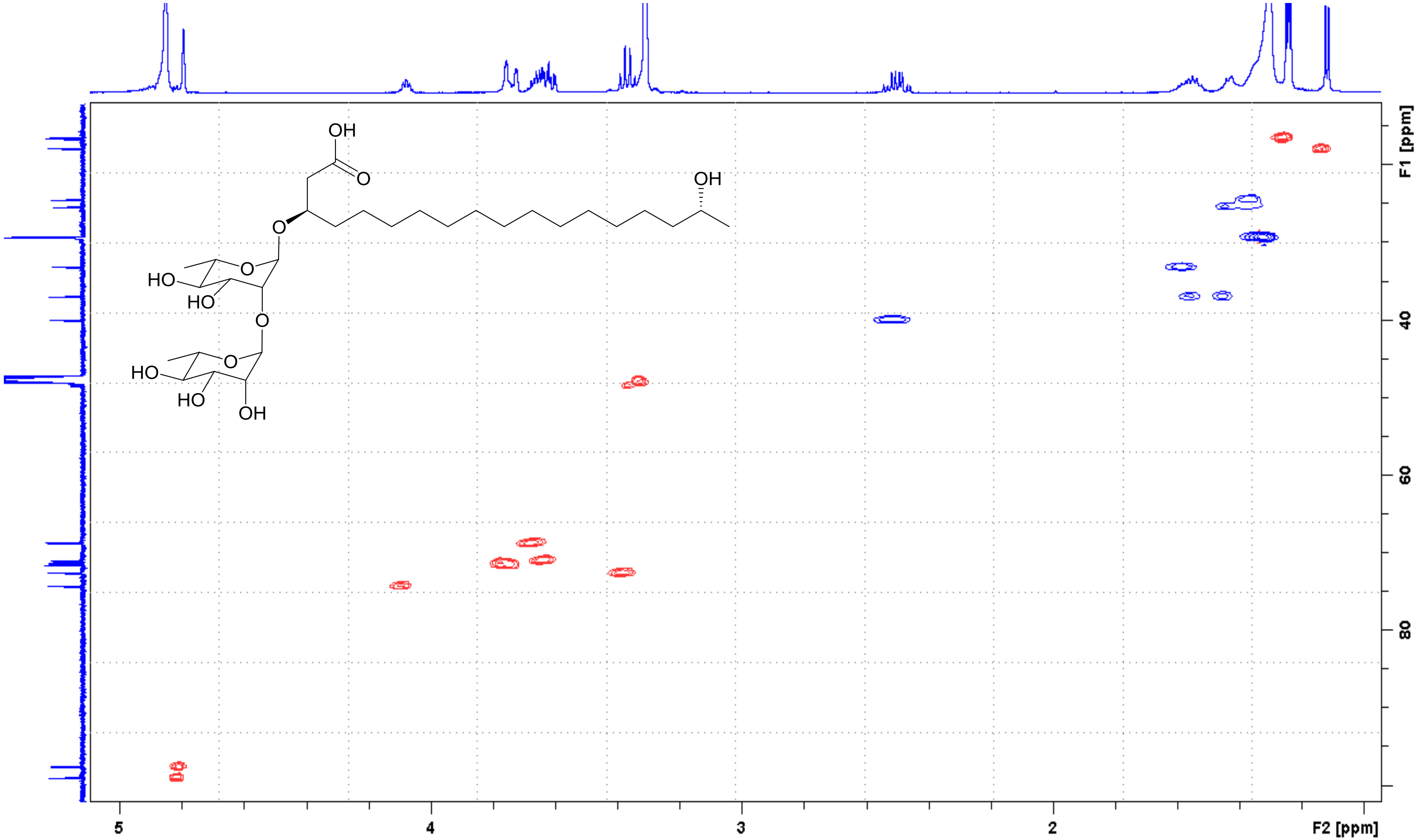

Figure S23. HSQC spectrum of dokdolipid C (**3**).

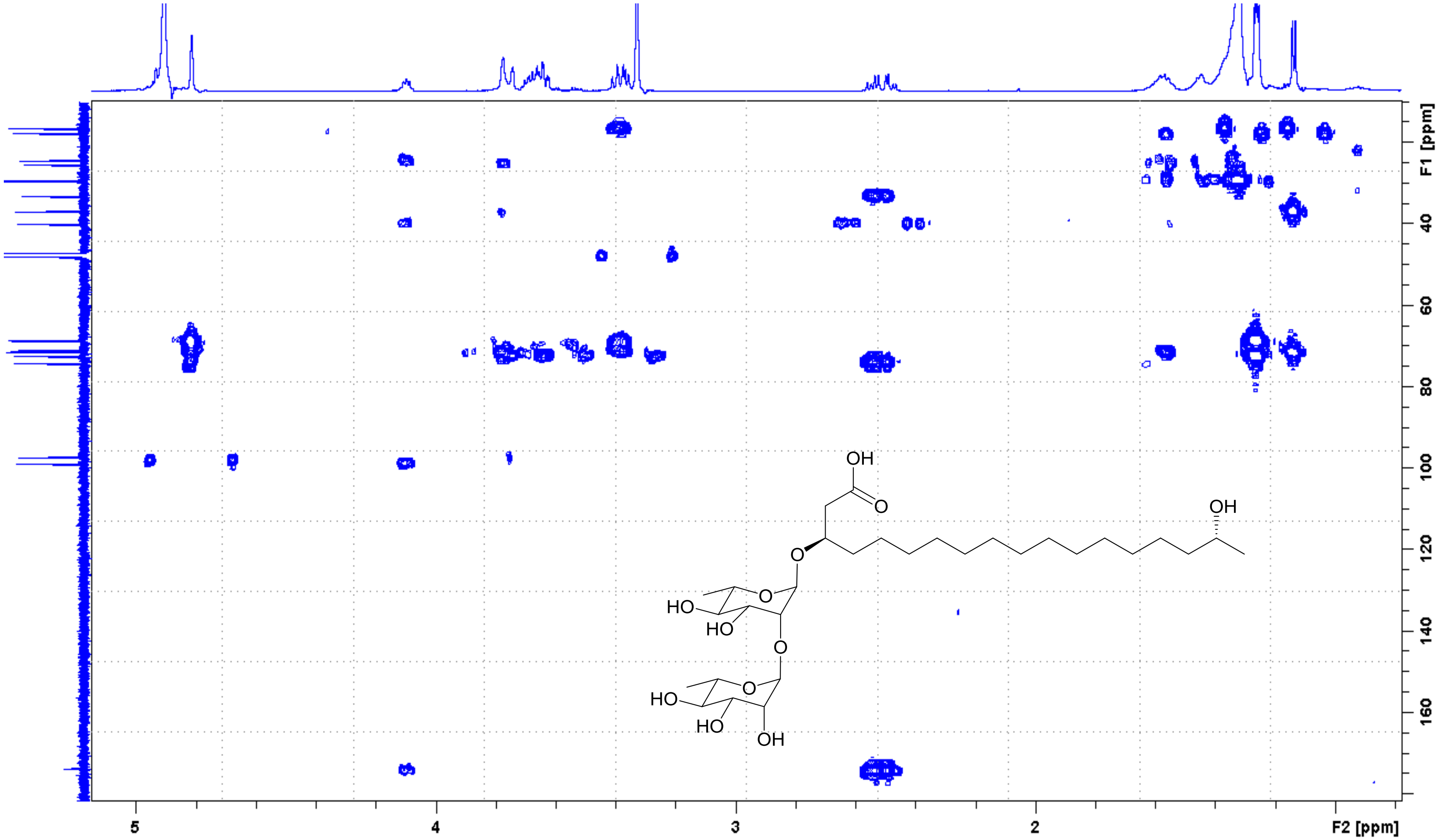

Figure S24. HMBC spectrum of dokdolipid C (**3**).

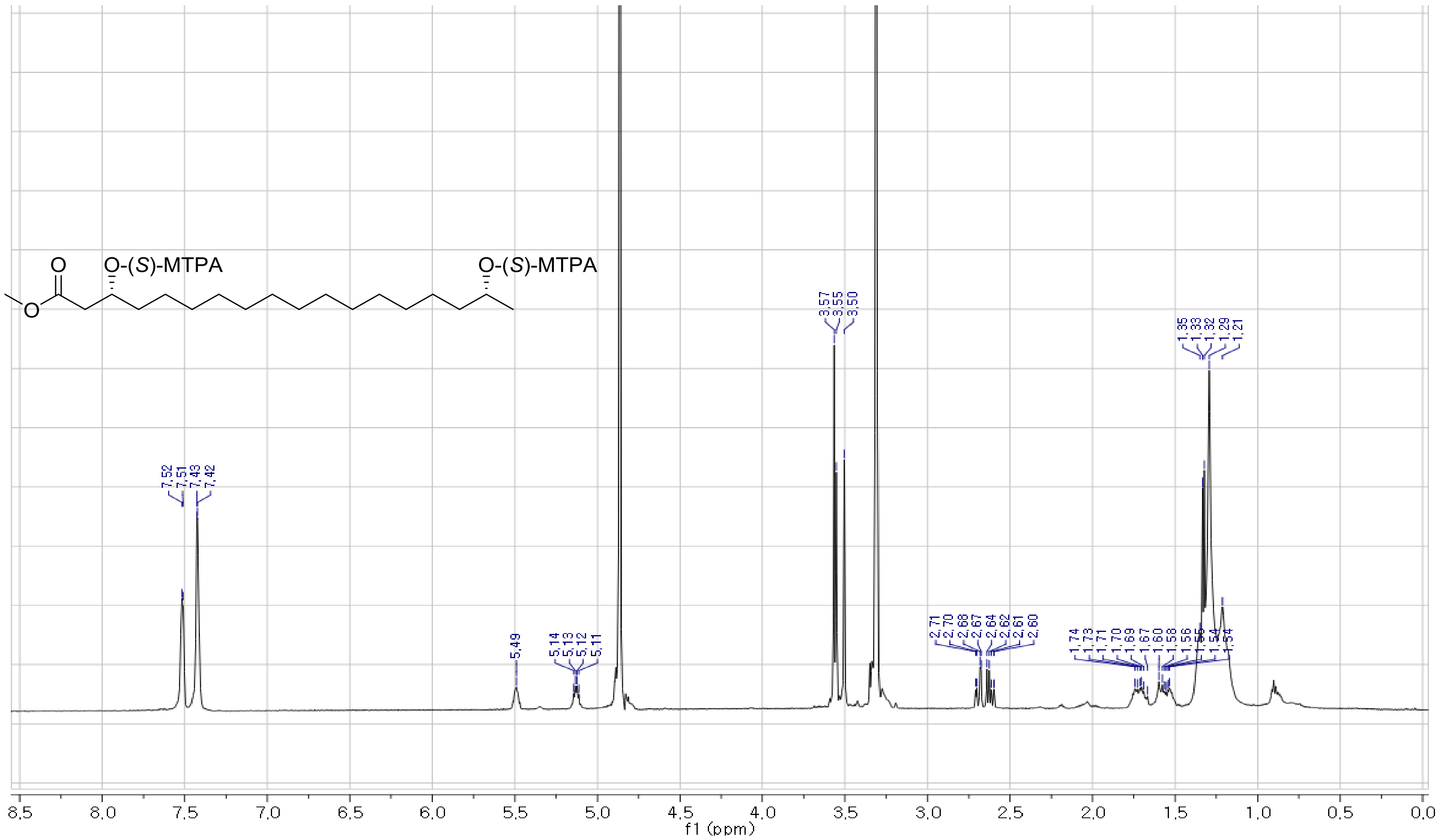

Figure S25. ^{1}H NMR spectrum of (*S*)-MTPA (**3a**) ester.

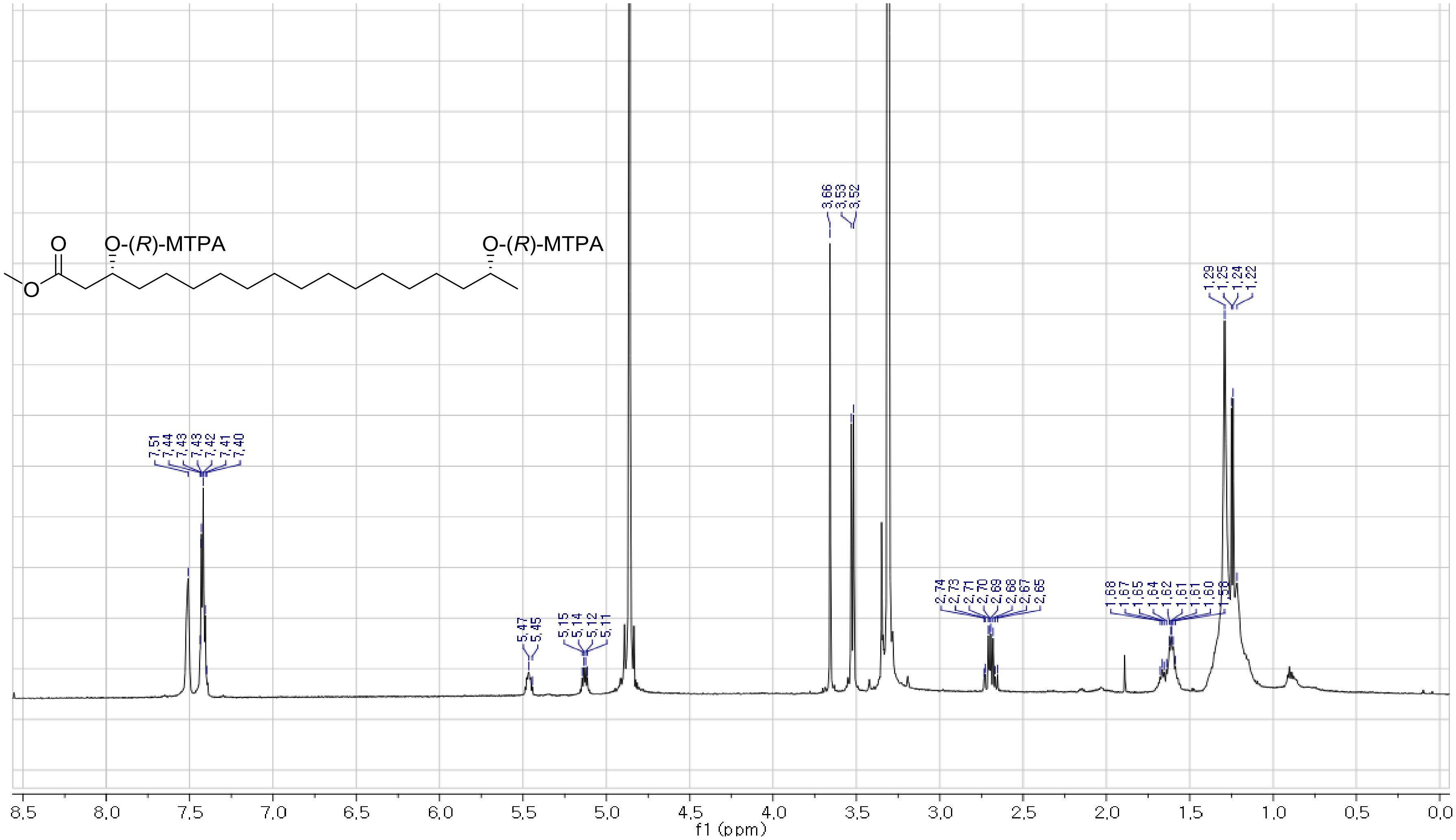

Figure S26. ^{1}H NMR spectrum of (*R*)-MTPA (**3b**) ester.
